# Genetic Diversity and Geographic Population Structure of Bovine *Neospora* *caninum* Determined by Microsatellite Genotyping Analysis

**DOI:** 10.1371/journal.pone.0072678

**Published:** 2013-08-06

**Authors:** Javier Regidor-Cerrillo, Francisco Díez-Fuertes, Alicia García-Culebras, Dadín P. Moore, Marta González-Warleta, Carmen Cuevas, Gereon Schares, Frank Katzer, Susana Pedraza-Díaz, Mercedes Mezo, Luis M. Ortega-Mora

**Affiliations:** 1 SALUVET, Animal Health Department, Faculty of Veterinary Sciences, Complutense University of Madrid, Madrid, Spain; 2 Consejo Nacional de Investigaciones Científicas y Técnicas (CONICET), Buenos Aires, Argentina; 3 Laboratory of Parasitology, Agricultural Research Center of Mabegondo (INGACAL), Mabegondo, A Coruña, Spain; 4 Friedrich-Loeffler-Institut, Federal Research Institute for Animal Health, Institute of Epidemiology, Greifswald–Insel Riems, Germany; 5 Moredun Research Institute, Bush Loan, Edinburgh, United Kingdom; Instituto de Higiene e Medicina Tropical, Portugal

## Abstract

The cyst-forming protozoan parasite *Neospora* *caninum* is one of the main causes of bovine abortion worldwide and is of great economic importance in the cattle industry. Recent studies have revealed extensive genetic variation among *N* *. caninum* isolates based on microsatellite sequences (MSs). MSs may be suitable molecular markers for inferring the diversity of parasite populations, molecular epidemiology and the basis for phenotypic variations in *N* *. caninum* , which have been poorly defined. In this study, we evaluated nine MS markers using a panel of 11 *N* *. caninum* -derived reference isolates from around the world and 96 *N* *. caninum* bovine clinical samples and one ovine clinical sample collected from four countries on two continents, including Spain, Argentina, Germany and Scotland, over a 10-year period. These markers were used as molecular tools to investigate the genetic diversity, geographic distribution and population structure of *N* *. caninum* . Multilocus microsatellite genotyping based on 7 loci demonstrated high levels of genetic diversity in the samples from all of the different countries, with 96 microsatellite multilocus genotypes (MLGs) identified from 108 *N* *. caninum* samples. Geographic sub-structuring was present in the country populations according to pairwise *F*_*ST*_. Principal component analysis (PCA) and Neighbor Joining tree topologies also suggested MLG segregation partially associated with geographical origin. An analysis of the MLG relationships, using eBURST, confirmed that the close genetic relationship observed between the Spanish and Argentinean populations may be the result of parasite migration (i.e., the introduction of novel MLGs from Spain to South America) due to cattle movement. The eBURST relationships also revealed genetically different clusters associated with the abortion. The presence of linkage disequilibrium, the co-existence of specific MLGs to individual farms and eBURST MLG relationships suggest a predominant clonal propagation for Spanish *N* *. caninum* MLGs in cattle.

## Introduction

*Neospora* *caninum* is a cyst-forming obligate intracellular parasite that is phylogenetically related to *Toxoplasma gondii* and has been recognised as a worldwide cause of neuromuscular disease in dogs and a major cause of abortion in cattle [1-3]. Advances in research concerning the *N* *. caninum* life cycle have confirmed that canids (dog, coyote, dingo and gray wolf) are intermediate and definitive hosts and that cattle and other ungulates, including sheep, goat, water buffalo, bison and deer, are natural intermediate hosts [1-3]. There is experimental evidence that the *N* *. caninum* -life cycle can be maintained by domestic dogs and cattle. Dogs can become infected by ingesting *N* *. caninum* -infected bovine tissues, and oocysts are shed in the faeces after sexual reproduction presumably in intestinal cells, which in turn can orally infect cattle [2-6]. Based on results observed previously for *T. gondii*, sexual crosses of *N* *. caninum* in canids could produce new isolates with distinct biological characteristics by recombination and reassortment of genetic material between two different parental genomes [7]. *N* *. caninum* can also be transmitted transplacentally to the foetus by a primary infection of a dam with oocysts during pregnancy (exogenous transmission) or the recrudescence of a chronic infection in the dam (endogenous transmission), causing fetopathy [8,9]. Endogenous vertical transmission is recognised as the main route of transmission in cattle [2,3,9], but it has been suggested that *N* *. caninum* infection is not likely to persist in the absence of horizontal transmission [10] and recent extensive serological analyses have demonstrated significant post-natal infection rates in cattle [11]. The extent to which each route of transmission contributes to bovine abortion can vary among herds, with different epidemiological and control implications [8]. In addition, a wildlife cycle for *N* *. caninum* has been described involving white-tailed deer ( *Odocoileus* *virginianus* ) and coyote ( *Canis* *latrans* ) as intermediate and definitive hosts, respectively [12,13].

Suitable genetic discriminatory methods for differentiating parasites at the strain or isolate levels are necessary to investigate the intra-species diversity and epidemiological and clinical aspects associated with neosporosis. Molecular genotyping techniques have been used to study the taxonomy, evolutionary mechanisms and phylogenetic relationships, population genetic structure and epidemiology of many organisms, including protozoan parasites [14-19]. These molecular methods could be used to establish the importance of domestic and sylvatic life cycles in neosporosis, clarify the role of horizontal transmission in spreading and perpetuating infection and abortion, determine the geographic distribution of isolates and evaluate the emergence of new *N* *. caninum* isolates with specific host preferences and virulence. These topics have been poorly investigated in *N* *. caninum* , likely due to a lack of suitable molecular tools. Several different studies have recently shown the discriminatory power of microsatellite (MS) markers to genetically identify *N* *. caninum* isolates from diverse hosts and geographic origins [20-25]. The use of MS markers to genotype *N* *. caninum* in clinical samples was facilitated by the development of nested-PCR [23,26]. Mini- and microsatellite markers have been broadly applied to investigate the epidemiology and population structure of diverse protozoan parasites, including *T. gondii* [19,27-30]. In the present study, we evaluated these MS markers used in multilocus as a genetic tool for large-scale population genetic analyses. Nine MS markers were evaluated using a panel of 11 *N* *. caninum* -derived reference isolates from around the world, 96 *N* *. caninum* bovine clinical samples and one ovine clinical sample collected from four countries, including Spain, Argentina, Germany and Scotland, on two continents over a 10-year period. These markers were used to investigate the genetic diversity of *N* *. caninum* at global and country level, geographic distribution of *N* *. caninum* genotypes and the genetic relatedness between country populations and population structure of *N* *. caninum* in cattle.

## Materials and Methods

### Parasites and Clinical DNA Samples

The *Neospora* DNA samples included in this study were obtained from isolates maintained in a laboratory and clinical samples from contaminated tissues collected during the necropsy of ruminant abortions, resulting in a total of 108 samples from various regions of the world (Figure S1, Table S1). The DNA samples from 11 *N* *. caninum* isolates that had been obtained from dogs with clinical *Neospora* infection (NC-1, Nc-Liv, Nc-Bahia and Nc-GER1), oocysts shed from a naturally infected dog ( *Hammondiaheydorni* -Berlin-1996; later referred to as *Hh*-Berlin isolate), bovine abortions, stillbirths or calves with neurological signs (Nc-SweB1, Nc-PV1, KBA1 and KBA2), a clinically healthy calf (Nc-Goiás 1) and a naturally infected sheep (named as Nc-Sheep) were used in this study. The majority of these isolates have been previously genotyped [20,31]. This worldwide group of samples served as the reference and out-group for comparison (Figure S1, Table S1).

Microsatellite multilocus genotype (MLG)-data of 10 *N* *. caninum* samples from asymptomatic-congenitally infected calves from different provinces in Spain (Nc-Spain1H-Nc-Spain10) collected from 2003–2006 were included in this study [21,32]. The remaining DNA samples were selected from infected tissues from abortions that were submitted to the Animal Health Department at the Complutense University of Madrid and the Agricultural Research Center of Mabegondo in Spain, the Instituto Nacional de Tecnología Agropecuaria of Balcarce in Argentina, the Institute of Epidemiology, Friedrich-Loeffler-Institut in Wusterhausen in Germany and the Moredun Research Institute in Scotland for routine diagnosis or further analysis. Prior to genotyping, the clinical DNA samples were analysed using nested *N* *. caninum* -specific ITS-1 PCR [21]. The PCR-positive samples were further analysed using MS genotyping. The DNA samples with five or more out of nine MS marker products were selected (Table S1).

#### Clinical samples of Spanish origin

A total of 53 DNA samples from the infected tissues of the aborted bovine foetuses collected primarily from Holstein-Friesian cattle between 2001 and 2010 were included in this study (Table S1). A total of 41 bovine samples were collected from an area of approximately 10,417 Km^2^ in Galicia (north-west Spain), which is one of the main cattle-producing regions in Spain; 11 bovine samples were collected from the Madrid, Navarra and Zaragoza provinces (central and north-east Spain) covering a geographic area of 343 Km^2^ and 7,097 Km^2^ for the central and north-east regions, respectively, and 1 ovine sample from Jaén province (southern Spain). These areas were located 285 to 556 Km apart in an area of 385,106 Km^2^. Of these, a total of 18 samples were genotyped in a previous study [26] (Figure S1, Table S1).

#### Clinical samples of Argentinean origin

A total of 16 DNA samples obtained from bovine foetuses between 2004 and 2008 were included in this study (Table S1). The foetuses originated from the humid pampas, the main cattle-producing area of Argentina, including the province of Buenos Aires, south of Santa Fe and Cordoba (Figure S1). Most of samples were collected from Buenos Aires province in an area of 149,146 Km^2^ and the farms for sampling were approximately 53 to 491 Km apart. The cattle were commercial beef (primarily Aberdeen Angus, Hereford and crossbreeds) and dairy (Holando Argentino and Jersey) breeds.

#### Clinical samples of German and Scottish origin

DNA samples obtained from nine bovine foetuses of the Holstein-Friesian breed collected in Germany between 2000 and 2009 were included in this study. The samples originated from different states, including Bavaria, Lower Saxony, North rhine-Westphalia, Schleswig-Holstein, Rhineland-Palatinate, Brandenburg and Hesse, comprising a geographic area of 233.657 Km^2^ and from farms located approximately 10 to 556 Km apart (Figure S1, Table S1).

A total of nine DNA samples were obtained in Scotland from bovine abortions between 2007 and 2009. The foetuses originated from south-west Scotland, in the Dumfries and Galloway region of 6.426 Km^2^ (Figure S1, Table S1). The farm and breed origin was not available.

### Multiplex Multilocus Nested PCR Microsatellite Genotyping

*N* *. caninum* genotyping was performed in ITS-1 PCR-positive clinical samples using the nine MS markers MS4, MS5, MS6A, MS6B, MS7, MS8, MS10, MS12 and MS21 that were previously described [20]. The targeted DNA sequences were first amplified by two rounds of multiplex PCRs for the MS4, MS5, MS7 and MS21 markers and the MS6A, MS6B, MS8, MS10 and MS12 markers, respectively. These multiplex PCRs were performed using approximately 200 ng of DNA as a template. The multiplex PCR-amplified products were diluted 1:5 in water and used for a second round of amplifications (nested-PCR) with separate internal primers for each marker. All of the external and internal primers, including the 6-FAM dye labelled reverse primers, and PCR conditions were previously described [26]. The size of the 6-FAM-labelled PCR products for all of the MSs was determined using a 48-capillary 3730 DNA analyzer (Applied Biosystems, Foster City, CA, USA) with Gene Scan-500 (LIZ) Size Standards (Applied Biosystems) at the Unidad Genómica del Parque Científico de Madrid and the GeneMapper1 V 3.5 Software (Applied Biosystems) as previously described [26].

MS7 and MS10 amplifications were also performed with non-labelled reverse primers for sequencing with the Big Dye Terminator v 3.1 Cycle Sequencing Kit (Applied Biosystems) and a 3730 DNA analyser (Applied Biosystems) at the Unidad Genómica del Parque Científico de Madrid. The sequences were analysed using BioEdit Sequence Alignment Editor v.7.0.1 (Copyright_ 1997–2004 Tom Hall, Ibis Therapeutics, Carlsbad, CA, USA).

Negative controls, including reactions without a template and extraction of bovine DNA negative for *Neospora* by ITS-1 PCR, were included in each round of PCR. In each round of amplifications, a *N* *. caninum* (Nc-Liv) reference isolate for which the sizes of the nine MS loci had been determined by sequencing was included as an internal control [20].

Allele assignment was performed according to the sizes determined by capillary electrophoresis and sequencing of the MS10 and MS7 markers. Allele designation in this study was performed according to the length of the variable repeat motifs identified in the MS sequences based on previous analyses [20,23,26] (Table S2).

### Data Analysis

The seven-loci MS profiles (MS6B and MS21 loci were excluded; see below) obtained from 108 samples infected with *N* *. caninum* were used for subsequent analyses.

#### Genetic and genotypic diversity within populations

We measured the allele number (A), allele frequency and allele richness (Ar) per locus in whole and separate parasite country populations using FSTAT v.2.9.3.2 ( [33]; http://www2.unil.ch/popgen/softwares/fstat.htm). The allele frequency illustrated the composition and distribution of alleles in whole and separate country populations. The Ar was corrected for unequal sample size by standardising the allelic richness to the smallest sample size in each dataset of 7 individuals. Genetic polymorphisms were measured by Nei’s unbiased genetic diversity within samples (He) using GDA software v.1.1 ( [34]; http://www.eeb.uconn.edu/people/plewis/software.php).

Genotypic diversity was calculated as the number of unique MLGs divided by the total number of individuals genotyped. Missing alleles were not considered in the genotype comparisons. Genotypic diversity was estimated for the entire population and within defined parasite populations.

#### Genetic distance (F-statistic) and linkage disequilibrium of the populations

The GDA package was used to calculate Nei’s genetic distance (*D*) between each group of *N* *. caninum* samples from different geographic locations [34,35]. The FSTAT package version 2.9.3.2 [33] was used for pairwise comparisons of the *F*_*ST*_ index based on Weir and Cockerham’s θ estimator [36] between each group of parasite populations. Significance testing was performed by comparing the values obtained after 10,000 random permutations of the dataset.

Multilocus linkage disequilibrium (LD) amongst seven-loci genotypes was assessed using a standardised index of association (I_A_^S^), implemented in the LIAN v 3.5 web interface ([37]; http://pubmlst.org/perl/mlstanalyse/mlstanalyse.pl?site=pubmlst&page=lian &referer=pubmlst.org). Because LIAN cannot analyse MLGs with missing data, only MLGs with complete allelic data for the seven markers were used for analyses. The presence of LD was assessed for whole and separate populations in: the complete dataset and a reduced dataset of unique MLGs, where duplicate MLGs (found in more than one sample) were only included once.

#### eBURST analysis

The eBURST software was used to explore the genetic relationships within entire dataset [38]. The eBURST software generates networks composed of MLGs represented as dots, whose diameter is proportional to the number of identical genotypes, linked to their single-locus (SLV- 6 shared loci of 7) and double-loci (DVL- 5 shared loci of 7) variants by lines. Because the missing data are not identified, only MLGs with complete allelic data were included.

#### Principal component analysis (PCA)

A seven-loci MLG dataset from each population was used for PCA [39]. Similarity comparisons between individual MLGs were performed using the allele-sharing coefficient as determined with the Excel Microsoft-Toolkit. The Microsoft Excel plug-in software GenAlEx6 [39] was used to generate a covariance matrix and perform PCA. Graphics showing the axes for each analysis were generated in Excel software.

#### Neighbor-Joining analysis

Genetic relatedness based on the proportion of shared alleles (DAS) and Cavalli-Sforza distances among the sample MLGs was calculated from the complete dataset by applying POPULATIONS v. 1.2.3.2 software (http://bioinformatics.org/~tryphon/populations/). An unrooted Neighbor-Joining (NJ) tree was constructed with the DAS matrix using POPULATIONS software. This analysis was performed with bootstrap values for 1,000 replicates. The NJ tree was visualised with FigTree v.1.3.1 (http://tree.bio.ed.ac.uk/software/figtree/).

## Results

### Allele Description: Chromosomal Location, Allelic Profile, *In Vitro* Stability of Microsatellite Markers and *N* *. caninum* Microsatellite Genotyping

The nine MS markers initially selected for *N* *. caninum* genotyping were located on six chromosomes (II, VIIa, VIII, IX, X and XII) based on a BLAST search in the ToxoDB database (http://toxodb.org/toxo/) (Table S2). Three MS pairs were located in the same chromosome: MS6A and MS6B (178 bp apart) on chromosome X, MS4 and MS5 (at >1,200 Kb) on chromosome IX and MS7 and MS21 (at >608 Kb) on chromosome VIIa (Table S2). Most of the MSs are located in inter-genic non-coding regions and only the MS10 marker is located in an intron sequence and the predicted sequence of the open reading frame NCLIV_036590 (Table S2).

The number and size of the alleles at each MS marker are also displayed in Table S2. The number of alleles ranged from 4 to 12 alleles at most of the loci. MS10 was the most polymorphic marker with 37 alleles. Only two alleles were identified for the MS21 marker, which was excluded from subsequent MLG-data analyses. Within the MS7 marker, two types of alleles were clearly defined by a single nucleotide polymorphism (SNP) -2 bp from the TA repetitive motif that results in an additional TA repeat for the two unique alleles identified as 9.1 and 10.1 (Table S2). Correlation between the repeat length and the number of alleles at a locus was determined for the MSs (*r* ^2^= 0.9288, *p*<0.0001 by linear regression analysis in GraphPad Prism v.5 software).

The influence of *in vitro* passages of the parasite on the stability of the repetitive motif for each MS was evaluated in a stock of *N* *. caninum* DNA from the NC-1 isolates routinely maintained in 7 different research laboratories. The unique MS variation among NC-1 samples was detected on the MS6B marker from a unique NC-1 sample lacking one AT repeat (Table S1).

The nine MS markers evaluated were initially used to genotype *N* *. caninum* in this study (Table S1). More than one allele was sporadically detected in <1% (7/937 loci examined) of the DNA extracted from the clinical bovine samples. Secondary alleles were recognised as peaks with similar fluorescence intensity than the predominant peak in the electropherogram traces, varying by a single repeat (Table S1). Because double alleles were limited to a unique locus per sample and the *N* *. caninum* stages in intermediate hosts are haploid, the duplicity in alleles most likely reflects a transitory shift in the MSs or artefact due to sample processing more than mixed infections. Thus, the dominant allele was selected to generate a single MLG at each sample (Table S1). The genotypic diversity in the complete dataset was high (0.89), with 96 distinct MLGs in the 108 *N* *. caninum* samples (Table S1). This high genotypic diversity was maintained even after the MS6B and MS21 alleles were excluded from the MLG analysis. Because the moderately polymorphic MS6B might act as a “dumb” allele in this *N* *. caninum* population and MS6B is physically linked to MS6A, MS6B was also excluded from further MLG analyses. Full seven-loci genotypes were obtained in 76% of the samples, as 15.7% and 8.3% of the samples were missing data for a single locus or two loci, respectively.

### MS and Genotypic Diversity in Bovine *N* *. caninum* Populations from Different Countries

The MLGs were grouped according to geographic origin (including MLGs from the worldwide reference group) to investigate the genetic diversity in bovine *N* *. caninum* from different regions. High levels of diversity were observed among the populations for most of the markers according to allele frequencies (Figure S2). Predominant allele frequencies and allele distributions, involving the identification of unique alleles in each geographical population, were distinct in each country (Figure S2). The number of alleles (A), allelic richness (Ar) and Nei’s genetic diversity (He) values per locus and geographic origin are shown in Table S3. The mean Ar and *He* were moderately-high in all of the population samples, varying from 4.23 to 5.95 and from 0.62 to 0.80, respectively (Table 1). These results indicate a high level of genetic diversity in bovine *N* *. caninum* in all of the regions studied. The worldwide reference population displayed the highest Ar and *He* values (Table 1), likely because the worldwide dataset includes MLGs for *N* *. caninum* from different hosts and diverse geographic origins, and therefore a higher genetic diversity would be expected in this *N* *. caninum* population.

**Table 1:** Genetic and genotypic diversity of *N* *. caninum* populations from different countries.

**Population**	**N** ^a^	**Number of alleles (A)^b^**	**Allele richness (Ar)^b^**	**Genetic diversity(*He*)^b^**	**Genotypic diversity^c^**
Worldwide*	11	6.42 ± 2.57	5.95 ± 2.17	0.80 ± 0.16	1.00 (11/11)^c^
**Argentina**	16	4.86± 2.61	4.26 ± 2.01	0.62 ± 0.31	1.00 (16/16)
**Scotland**	9	4.29 ± 1.50	4.23 ± 1.48	0.70 ± 0.11	0.89 (8/9)
**Germany**	9	4.86± 1.68	4.79 ± 2.05	0.76 ± 0.14	1.00 (9/9)
**Spain**	63	8.57 ± 4.28	5.01 ± 1.60	0.67 ± 0.21	0.83 (52/63)

^a^ N, Number of samples included for each population.

^b^ Average ± SD.

^c^ Genotypic diversity as the number of unique MLGs/total number of individuals genotyped.

^*^ Worldwide *N* *. caninum* isolates were used as the reference group.

Genotypic diversity was similarly high across all of the populations from different countries (Table 1). All of the individual samples from the worldwide, Argentinean and German populations had unique MLGs, and only two Scottish samples showed identical MLGs (SCOT-08-4 and SCOT-08-7). Of the 63 samples from the Spanish population, 52 different MLGs were found (0.83). Identical profiles in the Spanish population were essentially observed in samples originating from the same herd (Table S1), and minor genotypic diversity can be attributed to sampling bias. Only a single pair of identical MLGs (SP-05-GAL-31 and GER-05-6) was identified between the four country populations and worldwide out-group. These genotypic data suggested a degree of geographic sub-structuring between populations from different countries.

### Population Differentiation in Bovine *N* *. caninum* Populations from Different Countries

**Figure 1:**
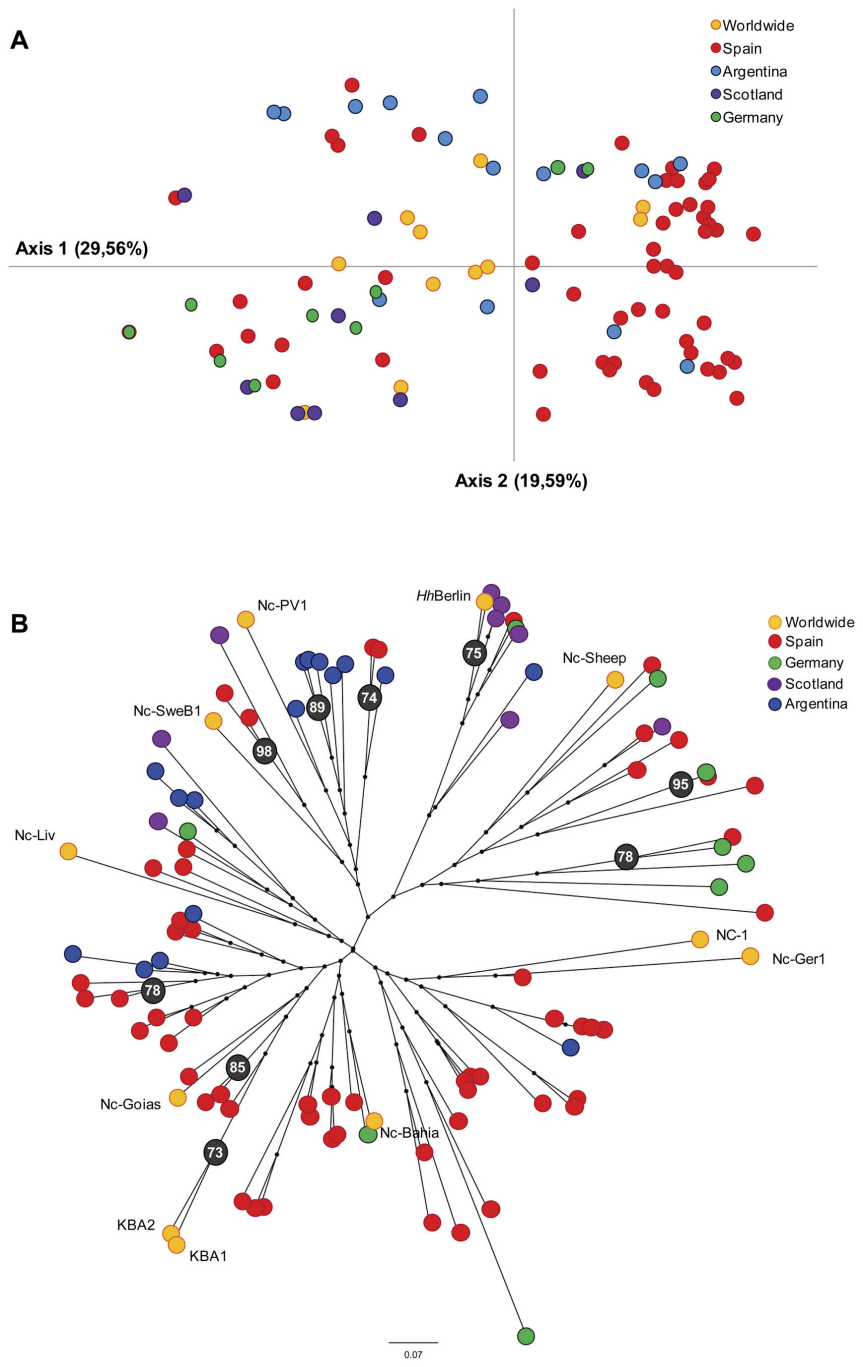
Clustering of *N* *. caninum* country populations based on Principal Component Analysis (PCA) and Neighbor Joining (NJ). (A) Graphics represent the genetic relationships between the MLGs from each country population based on a covariance matrix. Colours indicate geographic origin (see legend). The proportion of variation in the dataset for each axis is indicated in parentheses. (B) Unrooted NJ tree inferred from allele shared genetic distances. Each tip represents a single MLG. Woldwide MLGs are identified by isolate name. Colour of circles in terminal branches indicates geographic origin (see legend). Percentage bootstrap values were generated from 1,000 replicates. Bootstrap values ≥70% are shown in black circles. Scale bar represents branch lengths. NJ tree based in Cavalli-Sforza distances depicted similar topologies (data not shown).

Population differentiation was estimated between geographic regions using Nei’s genetic distance (*D*) and pairwise *F*_*ST*_ analysis (Table 2). Significant genetic differentiation was observed between the Spanish and German or Scottish populations, and between the Argentinean and German or Scottish populations (*D*> 0.4; pairwise *F*_*ST*_ values > 0.1). A lower level of genetic differentiation was observed between the Scottish and German populations (*D*= 0.3678; pairwise *F*_*ST*_= 0.0421). However, the pairwise *F*_*ST*_ value estimated between these populations was not significant, which is likely due to the limited sample size from both populations. Notably, when the Spanish and Argentinean populations were compared, minimal genetic differentiation was also observed (*D*= 0.2167; pairwise *F*_*ST*_= 0.0757). Geographical association of *N* *. caninum* MLGs was examined by overall structure PCA analysis (Figure 1A). The two axis account for 49% of variation and, although geographical populations are partially resolved, they showed a trend to be clustered into different quadrants, indicating geographical sub-structuring. Thus, the majority of German and Scottish MLGs were clustered in the far lower left quadrant, whereas the majority of Spanish MLGs were distributed in the far right quadrants together with several Argentinean MLGs. The remaining Argentinean MLGs were distributed in the higher right quadrant (Figure 1A). Clustering analysis of the MLGs using the NJ algorithm from DAS genetic distances also revealed different main branches grouping MLGs with mixed geographical origin (Figure 1B). Nevertheless, a more in depth evaluation showed a large number of Scottish, Argentinean and Spanish MLGs grouped within the particular branches. In addition, a mix of Argentinean and Spanish MLGs were associated in one of the main branches in accordance to PCA analysis. However bootstrap values in NJ dendrogram only showed significance towards the branch extremities, hampering to discern main genetically different groups associated with the geographical origin in *N* *. caninum* (Figure 1B). NJ nodes receiving statistical support by bootstrapping differentiated clusters of MLGs from the same region (e.g. KBA1 and KBA2) or herd origin, as well as of MLGs from distinct geographical locations. These results indicate that factors other than geographic origin and distance may influence the level of genetic differentiation observed between parasite populations. A clear MLG association with the date of collection of samples was not inferred from these clustering analyses (data not shown).

**Table 2:** Genetic differences between *N* *. caninum* populations: Matrix of Nei’s genetic distances (*D*) (below the principal diagonal) and pairwise *F*_*ST*_ analysis values (above the principal diagonal).

**Population**	Argentina	Scotland^#^	Germany^#^	Spain
Argentina		0.1081*	0.1338**	0.0757**
Scotland^#^	*0.4029*		0.0421	0.1280**
Germany^#^	*0.5788*	*0.3678*		0.1247*
Spain	*0.2167*	*0.5099*	*0.5523*	

^#^ Denotes small sample size (< 10).

* p< 0.005; **p< 0.001.

### Linkage Disequilibrium in Bovine *N* *. caninum* Populations

LD estimations were determined for each country population (Table 3). Multilocus analysis showed significant LD in the Argentinean, Spanish and Scottish populations (I_A_^S^> 0.1; p≤ 0.009). LD was slightly reduced in the Spanish population when the analysis was performed with unique MLGs, but with a higher level of statistical significance (I_A_^S^= 0.0872; p< 0.0001). These results suggest a non-random association between the MS loci in these parasite populations and support the possibility of genetic isolation. By contrast, significant LD was not detected among the German *N* *. caninum* MLGs. However, established LD estimations should be considered with caution due to the limited number of complete MLGs for some of the populations included in the analyses (<10). LD could be a consequence of the physical linkage of the polymorphic loci used for genotyping, such as MS4 and MS5. Complementary analyses of LD between all pairs of loci ruled out the influence of this MS pair in multilocus LD (data not shown).

**Table 3:** Linkage equilibrium and disequilibrium in *N* *. caninum* populations.

**Population**	**N^°a^**	**Index of association^b^ (I_A_^S^)**	**V_D_**	**L**	**V_D_ > L** ^c^	**P**
**Argentina**	10	0.1094	1.8455	1.5727	Y	0.009
**Scotland**	5^#^	0.2831	3.2111	2.322	Y	0.004
**Germany**	6^#^	-0.0062	1.0952	1.9524	N	0.21
**Spain**	50	0.1155*	2.1030	1.3922	Y	< 0.001

N^°^, Number of samples with a complete MS profile.

^b^ I_A_^S^, V_D_, L and P-values calculated by LIAN 3.5.

^c^ V_D_ > L indicates linkage disequilibrium (LD): Y, Yes; N, No.

^#^ Denotes small sample size (< 10).

^*^ I_A_^S^= 0.0872; p≤ 0.0001 for Spanish population when Lian 3.5 analysis was performed with unique MLGs.

**Figure 2:**
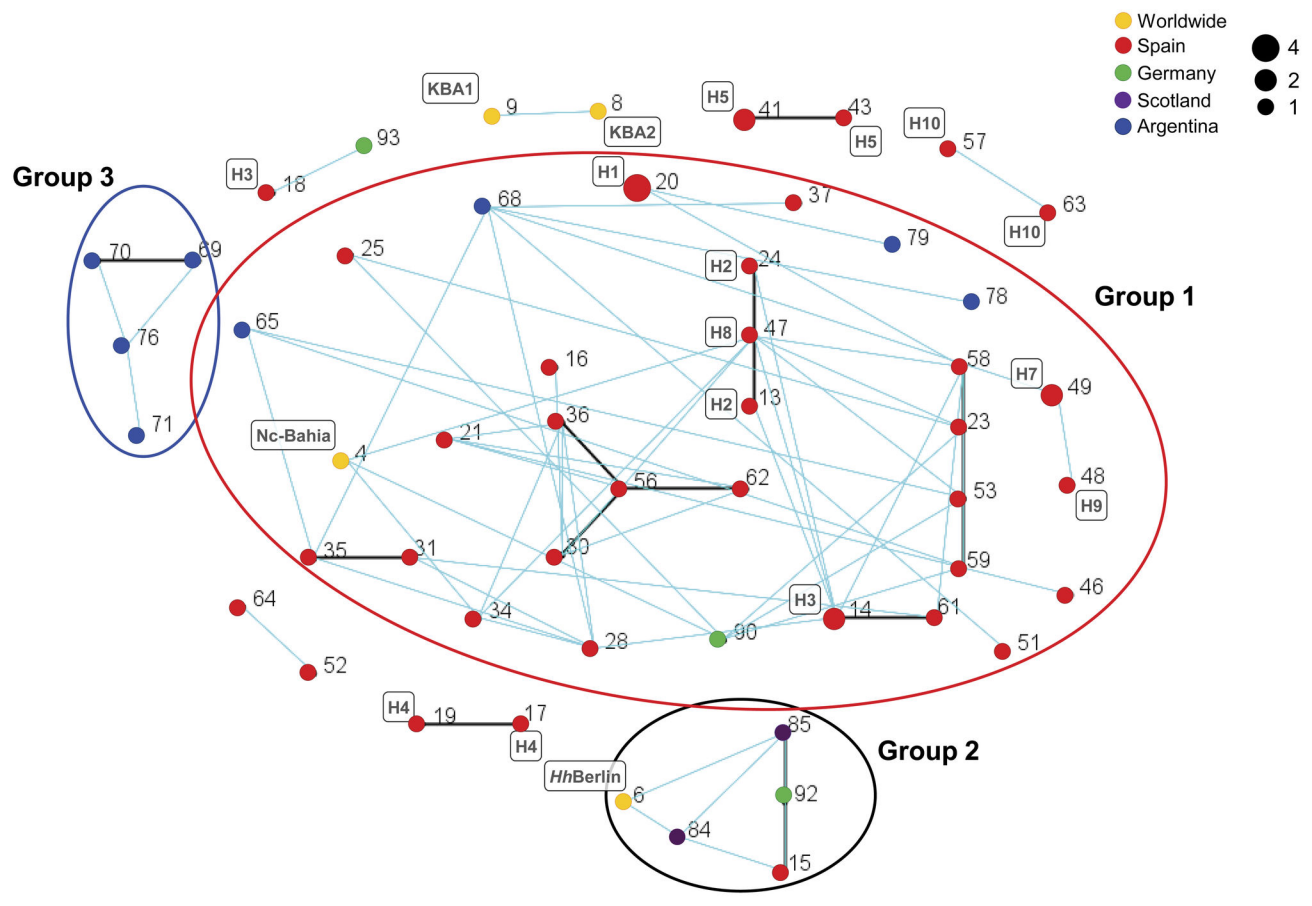
Relationships among the *N* *. caninum* MLGs (n = 82) estimated by eBURST analysis. Each complete MLG is represented by a dot and the genotype number assigned in Table S1 (Numbers 1-11 represent worldwide MLGs; 12-64 Spanish MLGs; 65-80 Argentinean MLGs; 81-88 Scottish MLGs; and 89-95 German MLGs). MLG dots were also coloured according to their geographical origin (see legend). The dot diameter is proportional to the number of samples with identical MLG (see legend). Single locus variants (SLV) are connected by black lines and double loci variants (DLV) by blue lines. MLGs clusters (n=9) are represented. Main MLGs clusters (n=3) are showed by clear circles. Number and name in the squares identifies the herd origin and worldwide isolates, respectively (see Table S1). Note that singletons were excluded from the snapshot representation (worldwide n=7; Spanish n=7; Argentinean n=2; Scottish n=3; and German n=2).

The relationship between MLGs was examined using eBURST software to explore the occurrence of SLVs and clonal clusters that may explain LD in country populations (Figure 2). When the whole dataset was visualised as a population snapshot, 4 main clonal SLV groups (with n≥ 3 MLGs) were detected, exclusively connecting Spanish MLGs. Interestingly, less stringent analysis considering DLVs revealed relationships among 53 (80%) MLGs from 59 samples, distributed within nine groups (Figure 2). Three main clusters (n≥ 3 MLGs) were recognised. The major cluster comprised 32 MLGs, primarily of Spanish (n= 26) and Argentinean (n= 4) origin with one German MLG and the Nc-Bahia (Brazilian) MLG (Figure 2, Group 1). The second group associated 5 MLGs distributed throughout Europe without apparent geographical connexion and grouping two Scottish MLGs, one German MLG and the *Hh*-Berlin (German) and Nc-Spain 5H (Spanish) MLGs (Figure 2, Group 2). Moreover, four Argentinean MLGs were associated in the third cluster (Figure 2, Group 3). In addition, 21 MLGs were singletons, lacking of association with other MLGs (data not shown). These eBURST findings suggest the presence of different clusters that comprise MLG variants that result more from high mutation rates than from recombination events. Notably, when the MLG origin at the herd level was recorded, a high number of clusters was shaped by MLGs as SLVs and DLVs from the same herd (e.g. MLG20 and MLG41-43) independently of time of collection, origin of the cattle and the circulation of genetically divergent MLGs at the same herd (e.g. Herd 3 and Herd 1-Table S1). These results indicate evidence of clonal propagation of *N* *. caninum* in these herds (Figure 2). Clonal expansion may also explain LD and resembles with the dynamics of *N* *. caninum* infection in domestic hosts. However, the moderate I_A_^S^ values obtained in the geographic populations and the complexity of the relationships between the MLGs in Group 1 did not discard some level of genetic exchange.

## Discussion

### Microsatellite Markers as Molecular Tools for *N* *. caninum* Genotyping

MS amplification of the clinical samples in this study was performed using nested-PCR developed by Pedraza-Díaz et al. [26], with slight modifications for the multiplexing of the first PCR reactions and increasing throughput. MS amplification was not achieved for all of the clinical samples and complete or nearly complete MS profiles were only obtained from approximately 50% of the ITS-1 PCR-positive DNA samples (data not shown), likely due to the fact that majority of the samples were extracted from aborted foetuses after mummification or autolysis in which the integrity of the DNA could be compromised [26]. Amplification failure led to a significant reduction in the sample size of the complete MLGs. For this reason, incomplete MS profiles were also included in some of these analyses.

Genotyping of a substantial number of samples also allowed us to define the suitability of these markers for the population genetic analysis of *N* *. caninum* . Novel alleles were found in most of the MS markers after analysis of the complete dataset, particularly for the most polymorphic marker MS10. This finding confirmed and increased the high discriminatory power for these markers established in previous studies [20,21,23,26]. The level of polymorphism in MSs is expected to be directly associated with the underlying mutation rate that is several orders of magnitude higher than that in other DNA sequences [40]. Several factors have been suggested to contribute to variations in the mutation rate of MSs such as the number of repeat motifs and the MS sequence [40,41], which were also associated with the level of polymorphism showed by MSs in this study. Nevertheless, the stability of these MS markers was confirmed when identical MS profiles were found for the NC-1 isolates collected from different laboratories. NC-1 has been maintained at the rapidly replicating tachyzoite stage in different cell cultures for undetermined successive passages and prolonged periods of time since its isolation in 1988 [2]. With regards to the principal variation (mutation) mechanism in MS sequences, it has been suggested to be DNA replication slippage, being the stepwise mutation model the simplest which assumes changes in only a single repeat unit [40-42]. Most of the alleles for each MS varied in length through the successive addition of one repeat unit following a normal distribution. In addition, MLGs sporadically showed double alleles that only differ by a single repeat unit, indicating a transient mutation/shift and a mutational pattern based on the stepwise mutation model in these MSs.

Multilocus analysis of MSs confirmed the great discriminatory power of these markers for the large-scale genotyping of *N* *. caninum* , as suggested in previous studies [20,21,23,26]. Extensive diversity was detected in *N* *. caninum* , with 96 MLGs identified in 108 *N* *. caninum* samples typed even when the multilocus analysis was limited to seven loci. High levels of genotypic diversity were also determined for the phylogenetically related *T. gondii* isolates when five MS markers were applied to French and French Guiana parasite populations or seven MSs were used to genotype 271 *T. gondii* isolates with different geographic origins [30,43]. These results strengthen the value of these MSs as robust, reproducible, highly polymorphic and stable molecular markers for epidemiological and population genetic studies of *N* *. caninum* at global and local scale.

### Multilocus Microsatellite Analysis Suggests Geographic Sub-structuring and a Predominant Clonal Population Structure of *N* *. caninum*

Similar average Ar and *He* values were found in populations originating from different countries, indicating high genetic diversity in *N* *. caninum* within each population. Similarly, high genotypic diversity was also observed for each *N* *. caninum* country population. Indeed, detection of identical MLGs was basically restricted to samples from individual herds confirming the high diversity of *N* *. caninum* on a smaller regional level.

Marked differences in the predominant allele frequencies and unique alleles specific to each population were observed, suggesting a high level of genetic differentiation between these geographic populations. Moderately high estimated pairwise *F*_*ST*_ and *D* values demonstrated a clear genetic separation between the Spanish and Argentinean populations, on one side, and the German and Scottish populations, on the other side. However, less genetic divergence was observed between the Spanish and Argentinean populations. Population segregation by NJ analysis was partially associated with the geographic origin by country, although dendrogram analysis based on these data was unstable. NJ analysis based on MLGs may failure in represent the complex population structure of *N* *. caninum* and detect the association between genetic divergence and geographical origin with accuracy [44]. When overall structure for *N* *. caninum* was also explored by PCA analysis, results revealed similar relationships for these country populations, showing a closer relationship between the Spanish and Argentinean populations and the German and Scottish populations. These results confirmed that there is a level of geographical sub-structuring between these populations. Considering the large distances and physical geographic barriers between these country populations - the Atlantic Ocean and Pyrenees Mountains - geographic differentiation is expected. However, genetic isolation by geographic distance cannot explain the minor genetic differences observed between the Spanish and Argentinean populations, and likely between the German and Scottish populations. When the relationships between individual MLGs were visualized by eBURST analysis, we observed two main clonal groups, clustering mainly Spanish and Argentinean MLGs (Figure 2, Group 1 and 3). Furthermore, the 40% of Argentinean MLGs were associated as DLVs to Spanish MLGs within the main clonal cluster (Figure 2, Group 1), demonstrating a close genetic relationship between these MLGs. Because a high level of polymorphism was determined by the MSs used in this study, “homoplasy” between Spanish and Argentinean *N* *. caninum* MLGs was discarded *a priori* [41]. A likely hypothesis for explaining these results is a recent MLG circulation, for example, migration of the parasite between country populations. Historical evidence indicates that Iberian cattle were introduced into South America by Spanish and Portuguese colonisers at the end of the 15th century. Iberian breeds were the founders of the adapted Creole breeds, unique South American bovines for more than 300 years until the introduction of new selected European and Zebu breeds [45,46]. By contrast to other apicomplexans, which have more mobile hosts (arthropod vectors and birds) as part of their life cycles, we can assume that the Argentinean *N* *. caninum* population may be engrossed by Spanish MLGs introduced via movement of cattle to generate a mixed population of *N* *. caninum* MLGs in Argentina. Domestic dogs, the definitive host of *N* *. caninum* , were simultaneously introduced with cattle and could also have been a vehicle for introducing *N* *. caninum* into South America or, more importantly, could have contributed to maintaining and spreading Spanish *N* *. caninum* MLGs inside South American herds. Therefore, the *N* *. caninum* clusters identified by eBURST could reflect mutation and genetic drift in geographically disconnected populations previously to introduction of new MLGs by host migration events.

Similar to the Spanish cattle breeds in South-America, the Holstein-Friesian breed originating from Holland-Germany was introduced worldwide into dairy herds during the second half of the 19th century and the first decades of the 20th century, including Spain [47]. As a result, *N* *. caninum* MLGs originating from Germany could have been globally distributed. However close relationships between German MLGs associated with MLGs from different geographic populations were only sporadically established by eBURST (MLG18-93). Additional investigations using a higher number of *N* *. caninum* samples, representing different European and American locations and a higher range of intermediate and definitive hosts, are needed to confirm this hypothesis. Moreover, new polymorphic markers based on genes and intron sequences must be evaluated in order to establish the history and geographic dissemination events for *N* *. caninum* .

The level of LD estimated for each population in this study also support parasite migration events. The observed LD implies low genetic exchange between parasite populations in the following scenarios: 1) “epidemic-ephemeral clonality”, 2) “cryptic biological speciation”, and 3) “clonal population structure”. LD might also reflect physical separation in either space or time or both, or the recent introduction of genetically different populations due to immigration and insufficient time for random mating or prolonged maintenance by clonal propagation [48,49]. This last scenario may be reflected by the significant LD (> 0.1) estimated in the Argentinean, Scottish and Spanish populations where genetically divergent *N* *. caninum* MLGs co-exist and are maintained by clonal propagation. This hypothesis is consistent with the results obtained from eBURST showing that different clonal clusters, some co-existing in individual herds, were described in the Spanish MLG population. Thus, the eBURST analysis suggests the clonal as the predominant form of propagation of *N* *. caninum* MLGs in the Spanish herd.

This clonal model is also compatible with the life cycle of *N* *. caninum* in domestic hosts, where the parasite is predominantly transmitted in cattle via vertical transmission routes after the reactivation of infection during the pregnancy or oral consumption of oocysts, likely after the inbreeding in the dog [23,50]. Nevertheless, the moderate I_A_^S^ values, the extensive genetic diversity showed by *N* *. caninum* with different clusters and the presence of singletons that are not associated to main cluster through eBURST analysis could be interpreted as the result of sexual recombination and genetic exchange. The life cycle of *T. gondii* and *N* *. caninum* allows clonal and sexual expansion in populations. Recent molecular studies in phylogenetically related *T. gondii* species have demonstrated different population patterns depending on the level of sexual recombination [19,43]. Recently, inbreeding in *T. gondii* has been associated with disease outbreaks due to the rapid amplification of a single clone into millions of oocysts and out-breeding as a potent mechanism to generate offspring with a range of phenotypic traits, including virulence [51]. The horizontal transmission route associated with infection from a common point source and likely inbreeding has been associated to epidemic outbreaks of abortion due to *N* *. caninum* in cattle herds [50]. Additional studies with larger intermediate and definitive host sample sizes and extensive MS genotyping would be valuable, particularly if these studies can be associated with additional information from serology and abortion data to provide insight into the importance of horizontal transmission, the level of sexual reproduction of the parasite and the frequency of out-breeding (genetic exchange), its influence on abortion rates, and the establishment of the actual population structure of *N* *. caninum* (clonal *vs.* panmitic).

A limitation of this study was the low number of *N* *. caninum* MLGs for some populations and representative values of the *N* *. caninum* samples from this parasite population. Despite increased efforts to obtain a representative number of *N* *. caninum* samples suitable for genotyping, the final parasite dataset was primarily limited to bovine abortion samples and *N* *. caninum* MLGs could be biased towards more virulent isolates in cattle. Samples from other domestic and wild intermediate and definitive hosts from different geographic regions should be included to establish the genetic diversity of *N* *. caninum* in these hosts and to investigate if there is host preference across *N* *. caninum* isolates and the global population structure of *N* *. caninum* . Additionally, the MS markers used in this study were physically distributed on 6 out of the 14 chromosomes of *N* *. caninum* . The seven MSs used in multilocus genotyping represent a suitable molecular tool for genetic studies in *N* *. caninum* according to the results obtained in this study. Future approaches should also be directed towards the identification of a new set of markers that represent the remaining 8 chromosomes and apicoplast DNA to improve this integrated method of *N* *. caninum* genotyping.

In summary, this work is the first extended study to infer the genetic diversity, geographic distribution and population structure in *N* *. caninum* from cattle using a set of seven MSs. The results demonstrate the suitability of these MSs as molecular tools to characterize the population structure of *N* *. caninum* . Multilocus MS analysis confirmed an extensive genetic diversity across *N* *. caninum* isolates from different countries and continents that is maintained at local level. Genetic differentiation between *N* *. caninum* geographic populations demonstrates that there is a degree of geographic sub-structuring by genetic isolation, although it could be influenced by parasite migration and host movement between countries. The results also support a predominant clonal propagation of *N* *. caninum* MLGs, at least in Spanish herds, but maybe under the presence of some degree of genetic exchange. Nevertheless, it has to be kept in mind that conclusions derived from the molecular analysis directly depend on the resolution of the markers and sampling used in the study. Extended analyses with an increased number of markers and representative samples from different hosts and geographic origins are needed to confirm the conclusions of this study.

## Supporting Information

Figure S1 **Geographic distribution of the *N* *. caninum* samples included in this study.** The expanded window shows the detailed geographic origins of the European *N* *. caninum* samples. Circle sizes represent the number of samples included for each country population as indicated by the number inside.
(TIF)

Figure S2 **Allele frequency distribution of the seven microsatellite loci used for genotyping each country population.** Fragment size and number of repetitive motifs were determined using Genescan software and sequencing. Frequencies were assessed based on the total number of samples included for each population. Allele lengths as number of repetitive motifs (or allele number assigned in Table S2) are indicated on the x-axis, while the y-axis shows the frequency of occurrence for each allele as a percentage.
(TIF)

Table S1 Multilocus microsatellite genotyping and origin of the *N* *. caninum* samples included in the study. (DOCX)

Table S2 Chromosomal location and summary of the alleles for each microsatellite marker. (DOCX)

Table S3 Genetic diversity in *N* *. caninum* by locus and geographic population (country). (DOCX)

## References

[1] Dubey JP, Schares G (2006) Diagnosis of bovine neosporosis. Vet Parasitol 140: 1-34. doi:10.1016/j.vetpar.2006.03.035. PubMed: 16730126. 16730126 10.1016/j.vetpar.2006.03.035

[2] Dubey JP, Schares G, Ortega-Mora LM (2007) Epidemiology and control of neosporosis and *Neospora caninum* . Clin Microbiol Rev 20: 323-367. doi:10.1128/CMR.00031-06. PubMed: 17428888. 17428888 10.1128/CMR.00031-06 PMC1865591

[3] Dubey JP, Schares G (2011) Neosporosis in animals--the last five years. Vet Parasitol 180: 90-108. doi:10.1016/j.vetpar.2011.05.031. PubMed: 21704458. 21704458 10.1016/j.vetpar.2011.05.031

[4] Gondim LF, Gao L, McAllister MM (2002) Improved production of *Neospora caninum* oocysts, cyclical oral transmission between dogs and cattle, and in vitro isolation from oocysts. J Parasitol 88: 1159-1163. doi:10.1645/0022-3395(2002)088[1159:IPONCO]2.0.CO;2. PubMed: 12537111. 12537111 10.1645/0022-3395(2002)088[1159:IPONCO]2.0.CO;2

[5] Gondim LF, McAllister MM, Anderson-Sprecher RC, Björkman C, Lock TF et al. (2004) Transplacental transmission and abortion in cows administered *Neospora caninum* oocysts. J Parasitol 90: 1394-1400. doi:10.1645/GE-359R. PubMed: 15715235. 15715235 10.1645/GE-359R

[6] McCann CM, McAllister MM, Gondim LF, Smith RF, Cripps PJ et al. (2007) *Neospora caninum* in cattle: Experimental infection with oocysts can result in exogenous transplacental infection, but not endogenous transplacental infection in the subsequent pregnancy. Int J Parasitol 37: 1631-1639. doi:10.1016/j.ijpara.2007.05.012. PubMed: 17624353. 17624353 10.1016/j.ijpara.2007.05.012

[7] Grigg ME, Bonnefoy S, Hehl AB, Suzuki Y, Boothroyd JC (2001) Success and virulence in *Toxoplasma* as the result of sexual recombination between two distinct ancestries. Science 294: 161-165. doi:10.1126/science.1061888. PubMed: 11588262. 11588262 10.1126/science.1061888

[8] Trees AJ, Williams DJ (2005) Endogenous and exogenous transplacental infection in *Neospora caninum* and *Toxoplasma gondii* . Trends Parasitol 21: 558-561. doi:10.1016/j.pt.2005.09.005. PubMed: 16223599. 16223599 10.1016/j.pt.2005.09.005

[9] Williams DJ, Hartley CS, Björkman C, Trees AJ (2009) Endogenous and exogenous transplacental transmission of *Neospora caninum* - how the route of transmission impacts on epidemiology and control of disease. Parasitology 136: 1895-1900. doi:10.1017/S0031182009990588. PubMed: 19691862. 19691862 10.1017/S0031182009990588

[10] French NP, Clancy D, Davison HC, Trees AJ (1999) Mathematical models of *Neospora caninum* infection in dairy cattle: Transmission and options for control. Int J Parasitol 29: 1691-1704. doi:10.1016/S0020-7519(99)00131-9. PubMed: 10608456. 10608456 10.1016/s0020-7519(99)00131-9

[11] Eiras C, Arnaiz I, Alvarez-García G, Ortega-Mora LM, Sanjuánl ML et al. (2011) *Neospora caninum* seroprevalence in dairy and beef cattle from the northwest region of Spain, Galicia. Prev Vet Med 98: 128-132. doi:10.1016/j.prevetmed.2010.10.014. PubMed: 21145605. 21145605 10.1016/j.prevetmed.2010.10.014

[12] Gondim LF (2006) *Neospora caninum* in wildlife. Trends Parasitol 22: 247-252. doi:10.1016/j.pt.2006.03.008. PubMed: 16616642. 16616642 10.1016/j.pt.2006.03.008

[13] Rosypal AC, Lindsay DS (2005) The sylvatic cycle of *Neospora caninum*: Where do we go from here? Trends Parasitol 21: 439-440. doi:10.1016/j.pt.2005.08.003. PubMed: 16098812. 16098812 10.1016/j.pt.2005.08.003

[14] Anderson TJ, Haubold B, Williams JT, Estrada-Franco JG, Richardson L et al. (2000) Microsatellite markers reveal a spectrum of population structures in the malaria parasite *Plasmodium falciparum* . Mol Biol Evol 17: 1467-1482. doi:10.1093/oxfordjournals.molbev.a026247. PubMed: 11018154. 11018154 10.1093/oxfordjournals.molbev.a026247

[15] Tanriverdi S, Grinberg A, Chalmers RM, Hunter PR, Petrovic Z et al. (2008) Inferences about the global population structures of *Cryptosporidium parvum* and *Cryptosporidium hominis* . Appl Environ Microbiol 74: 7227-7234. doi:10.1128/AEM.01576-08. PubMed: 18836013. 18836013 10.1128/AEM.01576-08 PMC2592928

[16] Llewellyn MS, Lewis MD, Acosta N, Yeo M, Carrasco HJ et al. (2009) *Trypanosoma cruzi* IIc: Phylogenetic and phylogeographic insights from sequence and microsatellite analysis and potential impact on emergent Chagas disease. PLOS Negl Trop Dis 3: e510. doi:10.1371/journal.pntd.0000510. PubMed: 19721699. 19721699 10.1371/journal.pntd.0000510 PMC2727949

[17] Ferreira GE, dos Santos BN, Dorval ME, Ramos TP, Porrozzi R et al. (2012) The genetic structure of *Leishmania infantum* populations in Brazil and its possible association with the transmission cycle of visceral leishmaniasis. PLOS ONE 7: e36242. doi:10.1371/journal.pone.0036242. PubMed: 22606248. 22606248 10.1371/journal.pone.0036242 PMC3350531

[18] Conrad MD, Gorman AW, Schillinger JA, Fiori PL, Arroyo R et al. (2012) Extensive genetic diversity, unique population structure and evidence of genetic exchange in the sexually transmitted parasite *Trichomonas vaginalis* . PLOS Negl Trop Dis 6: e1573. doi:10.1371/journal.pntd.0001573. PubMed: 22479659. 22479659 10.1371/journal.pntd.0001573 PMC3313929

[19] Su C, Khan A, Zhou P, Majumdar D, Ajzenberg D et al. (2012) Globally diverse *Toxoplasma gondii* isolates comprise six major clades originating from a small number of distinct ancestral lineages. Proc Natl Acad Sci U S A 109: 5844-5849. doi:10.1073/pnas.1203190109. PubMed: 22431627. 22431627 10.1073/pnas.1203190109 PMC3326454

[20] Regidor-Cerrillo J, Pedraza-Díaz S, Gómez-Bautista M, Ortega-Mora LM (2006) Multilocus microsatellite analysis reveals extensive genetic diversity in *Neospora caninum* . J Parasitol 92: 517-524. doi:10.1645/GE-713R.1. PubMed: 16883994. 16883994 10.1645/GE-713R.1

[21] Regidor-Cerrillo J, Gómez-Bautista M, Pereira-Bueno J, Aduriz G, Navarro-Lozano V et al. (2008) Isolation and genetic characterization of *Neospora caninum* from asymptomatic calves in Spain. Parasitology 135: 1651-1659. doi:10.1017/S003118200800509X. PubMed: 18980700. 18980700 10.1017/S003118200800509X

[22] Basso W, Herrmann DC, Conraths FJ, Pantchev N, Vrhovec MG et al. (2009) First isolation of *Neospora caninum* from the faeces of a dog from Portugal. Vet Parasitol 159: 162-166. doi:10.1016/j.vetpar.2008.10.025. PubMed: 19036520. 19036520 10.1016/j.vetpar.2008.10.025

[23] Basso W, Schares S, Bärwald A, Herrmann DC, Conraths FJ et al. (2009) Molecular comparison of *Neospora caninum* oocyst isolates from naturally infected dogs with cell culture-derived tachyzoites of the same isolates using nested polymerase chain reaction to amplify microsatellite markers. Vet Parasitol 160: 43-50. doi:10.1016/j.vetpar.2008.10.085. PubMed: 19084341. 19084341 10.1016/j.vetpar.2008.10.085

[24] Al-Qassab S, Reichel MP, Ivens A, Ellis JT (2009) Genetic diversity amongst isolates of *Neospora caninum*, and the development of a multiplex assay for the detection of distinct strains. Mol Cell Probes 23: 132-139. doi:10.1016/j.mcp.2009.01.006. PubMed: 19496247. 19496247 10.1016/j.mcp.2009.01.006 PMC3820043

[25] Al-Qassab S, Reichel MP, Ellis J (2010) A second generation multiplex PCR for typing strains of *Neospora caninum* using six DNA targets. Mol Cell Probes 24: 20-26. doi:10.1016/j.mcp.2009.08.002. PubMed: 19683051. 19683051 10.1016/j.mcp.2009.08.002

[26] Pedraza-Díaz S, Marugán-Hernández V, Collantes-Fernández E, Regidor-Cerrillo J, Rojo-Montejo S et al. (2009) Microsatellite markers for the molecular characterization of *Neospora caninum*: Application to clinical samples. Vet Parasitol 166: 38-46. doi:10.1016/j.vetpar.2009.07.043. PubMed: 19720464. 19720464 10.1016/j.vetpar.2009.07.043

[27] Ajzenberg D, Bañuls AL, Tibayrenc M, Dardé ML (2002) Microsatellite analysis of *Toxoplasma gondii* shows considerable polymorphism structured into two main clonal groups. Int J Parasitol 32: 27-38. doi:10.1016/S0020-7519(01)00301-0. PubMed: 11796120. 11796120 10.1016/s0020-7519(01)00301-0

[28] Ajzenberg D, Bañuls AL, Su C, Dumètre A, Demar M et al. (2004) Genetic diversity, clonality and sexuality in *Toxoplasma gondii* . Int J Parasitol 34: 1185-1196. doi:10.1016/j.ijpara.2004.06.007. PubMed: 15380690. 15380690 10.1016/j.ijpara.2004.06.007

[29] Mercier A, Devillard S, Ngoubangoye B, Bonnabau H, Bañuls AL et al. (2010) Additional haplogroups of *Toxoplasma gondii* out of Africa: Population structure and mouse-virulence of strains from Gabon. PLOS Negl Trop Dis 4: e876. doi:10.1371/journal.pntd.0000876. PubMed: 21072237. 21072237 10.1371/journal.pntd.0000876 PMC2970538

[30] Mercier A, Ajzenberg D, Devillard S, Demar MP, de Thoisy B et al. (2011) Human impact on genetic diversity of *Toxoplasma gondii*: Example of the anthropized environment from French Guiana. Infect Genet Evol 11: 1378-1387. doi:10.1016/j.meegid.2011.05.003. PubMed: 21600306. 21600306 10.1016/j.meegid.2011.05.003

[31] Garcia-Melo DP, Regidor-Cerrillo J, Ortega-Mora Miguel L, Collantes-Fernandez E, Ferreira de Oliveira VS, et al (2009) Isolation and biological characterisation of a new isolate of *Neospora caninum* from an asymptomatic calf in Brazil. Acta Parasitol 54: 180-185. doi:10.2478/s11686-009-0018-2.

[32] Rojo-Montejo S, Collantes-Fernández E, Regidor-Cerrillo J, Alvarez-García G, Marugan-Hernández V et al. (2009) Isolation and characterization of a bovine isolate of *Neospora caninum* with low virulence. Vet Parasitol 159: 7-16. doi:10.1016/j.vetpar.2008.10.009. PubMed: 19027235. 19027235 10.1016/j.vetpar.2008.10.009

[33] Goudet J (1995) FSTAT (version 1.2): A computer program to calculate F-statistics. J Hered 86: 485-486.

[34] Lewis PO, Zaykin D (2001) Genetic data analysis: Computer program for the analysis of allelic data. Available: http://www.eeb.uconn.edu/people/plewis/software.php.

[35] Nei M (1987) Molecular evolutionary genetics. New York: Columbia University Press. 512 pp.

[36] Weir BS, Cockerham CC (1984) Estimating F-statistics for the analysis of population-structure. Evolution 38: 1358-1370. doi:10.2307/2408641. 28563791 10.1111/j.1558-5646.1984.tb05657.x

[37] Haubold B, Hudson RR (2000) LIAN 3.0: Detecting linkage disequilibrium in multilocus data. Bioinformatics 16: 847-848. doi:10.1093/bioinformatics/16.9.847. PubMed: 11108709. 11108709 10.1093/bioinformatics/16.9.847

[38] Feil EJ, Li BC, Aanensen DM, Hanage WP, Spratt BG (2004) eBURST: Inferring patterns of evolutionary descent among clusters of related bacterial genotypes from multilocus sequence typing data. J Bacteriol 186: 1518-1530. doi:10.1128/JB.186.5.1518-1530.2004. PubMed: 14973027. 14973027 10.1128/JB.186.5.1518-1530.2004 PMC344416

[39] Peakall R, Smouse PE (2006) GENALEX 6: Genetic analysis in excel. population genetic software for teaching and research. Mol Ecol Notes 6: 288-295. doi:10.1111/j.1471-8286.2005.01155.x. 10.1093/bioinformatics/bts460 PMC3463245 22820204

[40] Ellegren H (2000) Microsatellite mutations in the germline: Implications for evolutionary inference. Trends Genet 16: 551-558. doi:10.1016/S0168-9525(00)02139-9. PubMed: 11102705. 11102705 10.1016/s0168-9525(00)02139-9

[41] Estoup A, Jarne P, Cornuet JM (2002) Homoplasy and mutation model at microsatellite loci and their consequences for population genetics analysis. Mol Ecol 11: 1591-1604. doi:10.1046/j.1365-294X.2002.01576.x. PubMed: 12207711. 12207711 10.1046/j.1365-294x.2002.01576.x

[42] Schlötterer C (2000) Evolutionary dynamics of microsatellite DNA. Chromosoma 109: 365-371. doi:10.1007/s004120000089. PubMed: 11072791. 11072791 10.1007/s004120000089

[43] Lehmann T, Marcet PL, Graham DH, Dahl ER, Dubey JP (2006) Globalization and the population structure of *Toxoplasma gondii* . Proc Natl Acad Sci U S A 103: 11423-11428. doi:10.1073/pnas.0601438103. PubMed: 16849431. 16849431 10.1073/pnas.0601438103 PMC1544101

[44] Pritchard JK, Stephens M, Donnelly P (2000) Inference of population structure using multilocus genotype data. Genetics 155: 945-959. PubMed: 10835412. 10835412 10.1093/genetics/155.2.945 PMC1461096

[45] Giovambattista G, Ripoli MV, Peral-Garcia P, Bouzat JL (2001) Indigenous domestic breeds as reservoirs of genetic diversity: The Argentinean creole cattle. Anim Genet 32: 240-247. doi:10.1046/j.1365-2052.2001.00774.x. PubMed: 11683709. 11683709 10.1046/j.1365-2052.2001.00774.x

[46] Lirón JP, Peral-García P, Giovambattista G (2006) Genetic characterization of Argentine and Bolivian creole cattle breeds assessed through microsatellites. J Hered 97: 331-339. doi:10.1093/jhered/esl003. PubMed: 16793865. 16793865 10.1093/jhered/esl003

[47] Sanchez-Belda A (2002) Razas Ganaderas Españolas Bovinas (Spanish Cattle Breeds). Madrid: FEAGAS-MAPA. 354 p.

[48] Tibayrenc M (1998) Genetic epidemiology of parasitic protozoa and other infectious agents: The need for an integrated approach. Int J Parasitol 28: 85-104. doi:10.1016/S0020-7519(97)00180-X. PubMed: 9504337. 9504337 10.1016/s0020-7519(97)00180-x

[49] Tibayrenc M, Ayala FJ (2002) The clonal theory of parasitic protozoa: 12 years on. Trends Parasitol 18: 405-410. doi:10.1016/S1471-4922(02)02357-7. PubMed: 12377258. 12377258 10.1016/s1471-4922(02)02357-7

[50] Basso W, Schares S, Minke L, Bärwald A, Maksimov A et al. (2010) Microsatellite typing and avidity analysis suggest a common source of infection in herds with epidemic *Neospora caninum*-associated bovine abortion. Vet Parasitol 173: 24-31. doi:10.1016/j.vetpar.2010.06.009. PubMed: 20609521. 20609521 10.1016/j.vetpar.2010.06.009

[51] Wendte JM, Miller MA, Lambourn DM, Magargal SL, Jessup DA et al. (2010) Self-mating in the definitive host potentiates clonal outbreaks of the apicomplexan parasites *Sarcocystis neurona* and *Toxoplasma gondii* . PLOS Genet 6: e1001261 PubMed: 21203443. 21203443 10.1371/journal.pgen.1001261 PMC3009688

