# Supplementary figures and images for: Genetic Diversity and Geographic Population Structure of Bovine Neospora caninum Determined by Microsatellite Genotyping Analysis
Source: PLoS One. 2013 Aug 6;8(8):e72678. doi: 10.1371/journal.pone.0072678 (PMC3735528; doi:10.1371/journal.pone.0072678)

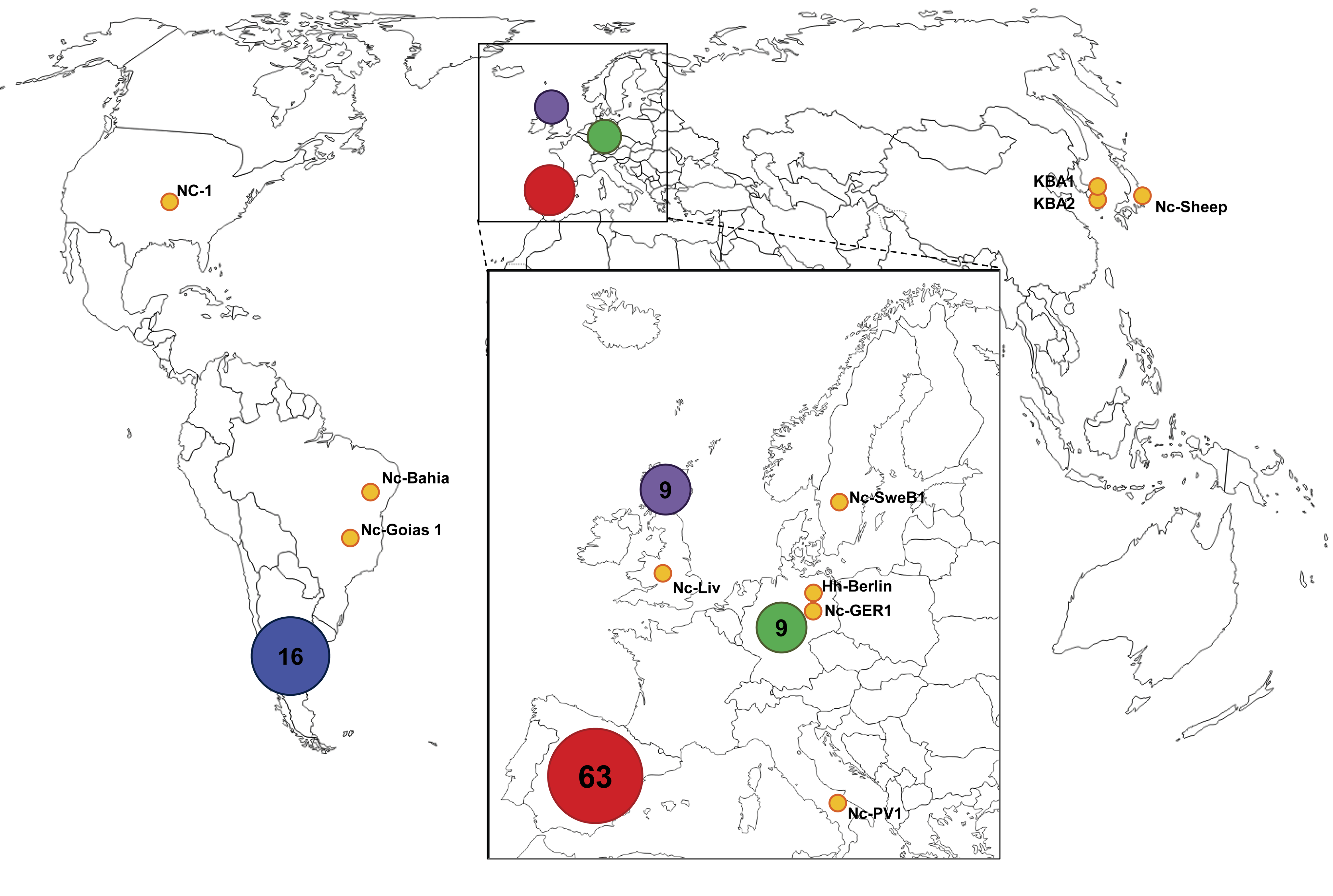

Supplement: Figure S1 — Geographic distribution of the N . caninum samples included in this study. The expanded window shows the detailed geographic origins of the European N . caninum samples. Circle sizes represent the number of samples included for each country population as indicated by the number inside. (TIF) [file pone.0072678.s001.tif]
