# Supplementary material for: Genetic Diversity and Geographic Population Structure of Bovine Neospora caninum Determined by Microsatellite Genotyping Analysis
Source: PLoS One. 2013 Aug 6;8(8):e72678. doi: 10.1371/journal.pone.0072678 (PMC3735528; doi:10.1371/journal.pone.0072678)
Supplement: Table S1 — (DOCX) [file pone.0072678.s003.docx]

**Supplementary Table 1:** Multilocus microsatellite genotyping and origin of the *N. caninum* samples included in the study.

| ***SAMPLE ID*** | | ***GEOGRAPHIC ORIGIN*** | | ***HOST SAMPLED*** | ***MICROSATELLITE GENOTYPE^c^*** | | | | | | | | |  | **Reference** |
| --- | --- | --- | --- | --- | --- | --- | --- | --- | --- | --- | --- | --- | --- | --- | --- |
| Original ID^a^ | Assigned ID^b^ | Country | Province |  | MS4 | MS5 | MS6A | MS6B | MS7 | MS8 | MS10 | MS12 | MS21 | **MLG^e^** |  |
| NC-1 | NC-1 | USA |  | Dog | 12 | 12 | 12 | 17^§^ | 16 | 16 | 7.12.9 | 16 | 6 | **1** | This study |
| NC-LIV | Nc-Liv | Great Britain |  | Dog | 15 | 9 | 15 | 12 | 14 | 17 | 6.26.10 | 16 | 6 | **2** | [1] |
| Nc-SweB1 | Nc-SweB | Sweden |  | Cattle (HF)^#^ | 13 | 13 | 12 | 14 | 13 | 14 | 8.23.8 | 16 | 6 | **3** | - |
| Nc-Bahia | Nc-Bahia | Brazil |  | Dog | 13 | 13 | 15 | 12 | 9.1 | 14 | 5.14.9 | 16 | 6 | **4** | - |
| Nc-PV1 | Nc-PV1 | Italy |  | Cattle (HF) | 14 | 11 | 19 | 12 | 14 | 16 | 6.21.10 | 16 | 6 | **5** | - |
| *H. h*-Berlin | Hh-Berlin | Germany | Berlin | Dog | 11 | 10 | 13 | 11 | 9.1 | 19 | 6.17.8 | 17 | 6 | **6** | - |
| Nc-GER1 | Nc-GER1 | Germany | Northrhine-Westphalia | Dog | 12 | 12 | 14 | 12 | 13 | 14 | 6.25.9 | 17 | 6 | **7** | - |
| KBA1 | KBA1 | Korea |  | Cattle (HF) | 15 | 14 | 14 | 12 | 11 | 15 | 6.22.9 | 16 | 6 | **8** | - |
| KBA2 | KBA2 | Korea |  | Cattle (HF) | 15 | 17 | 14 | 12 | 11 | 15 | 6.23.10 | 16 | 6 | **9** | - |
| Nc-Sheep | Nc-Sheep | Japan |  | Sheep | 10 | 9 | 15 | 13 | 11 | 13 | 6.24.8 | 15 | 6 | **10** | - |
| Nc-Goias | Nc-Goias | Brazil |  | Cattle (HF) | 13 | 18 | 14 | 12 | 9.1 | 12 | 6.16.9 | 16 | 6 | **11** | - |
| Nc-Spain1H | SP-03-MAD-NcSp1H | Spain | Madrid^H1^ | Cattle (HF) | 13 | 9 | 18 | 12 | 13 | 16 | 6.21.10 | 16 | 6 | **12** | [2] |
| Nc-Spain2H | SP-03-ZAR-NcSp2H | Spain | Zaragoza^H2^ | Cattle (HF) | 13 | 15 | 13 | 12 | 9.1 | 14 | 6.13.9 | 16 | 6 | **13** | - |
| Nc-Spain3H | SP-03-NAV-NcSp3H | Spain | Navarra^H3^ | Cattle (HF) | 13 | 15 | 14 | 11 | 9.1 | 14 | 6.14.9 | 16 | 6 | **14** | - |
| Nc-Spain4H | SP-03-NAV-NcSp4H | Spain | Navarra^H3^ | Cattle (HF) | 13 | 15 | 14 | 11 | 9.1 | 14 | 6.14.9 | 16 | 6 | **14** | - |
| Nc-Spain5H | SP-03-LEON-NcSp5H | Spain | León | Cattle (HF) | 11 | 11 | 13 | 11 | 9.1 | 16 | 6.16.8 | 17 | 6 | **15** | - |
| Nc-Spain6 | SP-03-VAS-NcSp6 | Spain | País Vasco | Cattle (HF) | 15 | 17 | 15 | 13 | 9.1 | 12 | 6.13.9 | 16 | 6 | **16** | - |
| Nc-Spain7 | SP-03-NAV-NcSp7 | Spain | Navarra^H4^ | Cattle (HF) | 13 | 10 | 13 | 11 | 10 | 16 | 6.23.10 | 16 | 5 | **17** | - |
| Nc-Spain8 | SP-03-NAV-NcSp8 | Spain | Navarra^H3^ | Cattle (HF) | 15 | 15 | 13 | 12 | 18 | 15 | 6.18.10 | 15 | 6 | **18** | - |
| Nc-Spain9 | SP-03-NAV-NcSp9 | Spain | Navarra^H4^ | Cattle (HF) | 13 | 10 | 13 | 11 | 10 | 16 | 6.21.10 | 16 | 5 | **19** | - |
| Nc-Spain10 | SP-06-MAD-NcSp10 | Spain | Madrid^H1^ | Cattle (HF) | 13 | 9 | 18 | 12 | 13 | 16 | 6.21.10 | 16 | 6 | **12** | - |
| 05-CT17 | SP-05-MAD-1 | Spain | Madrid^H1^ | Cattle (HF) | 12 | 15(I)/7* | 17 | 12 | 9.1 | 12 | 6.14.9 | 16 | 6 | **20** | - |
| 03-CF8 | SP-03-MAD-2 | Spain | Madrid | Cattle (HF) | 14 | 18 | 15 | 12 | 9.1 | 14 | 6.14.9 | 16 | 6 | **21** | [3] |
| 03-CF9 | SP-03-MAD-3 | Spain | Madrid^H1^ | Cattle (HF) | 12 | NA | 17 | 12 | NA | 12 | 6.14.9 | 16 | 6 | **20** | - |
| 03-Cf15B/Cf13-14 | SP-03-GAL-4 | Spain | Lugo | Cattle (HF) | 13 | 13 | 13 | 12 | 9.1 | 12 | NA | 15 | 6 | **22** | - |
| 05-Cf18 | SP-05-MAD-5 | Spain | Madrid^H1^ | Cattle (HF) | 12 | 15 | 17 | 12 | 9.1 | 12 | 6.14.9 | 16 | 6 | **20** | - |
| 05-CF21 | SP-05-MAD-6 | Spain | Madrid | Cattle (HF) | 13 | 15 | 15 | 11 | 9.1 | 12 | 6.14.8 | 16 | 6 | **23** | - |
|  | SP-03-ZAR-7 | Spain | Zaragoza^H2^ | Cattle (HF) | 13 | 15 | 12(I)/13* | 12 | 9.1 | 14 | 6.13.9 | 16 | 6 | **24** | - |
| OVINE 89 | SP-10-JAEN-8 | Spain | Jaen | Sheep | 13 | 13 | 15 | 12 | 9.1 | 12 | 6.14.8 | 15 | 6 | **25** | [4] |
|  | SP-08-MAD-9 | Spain | Madrid^H1^ | Cattle (HF) | 12 | 15 | NA | NA | 9.1 | 12 | 6.14.9 | 16 | 6 | **20** | This study |
|  | SP-08-MAD-10 | Spain | Madrid^H1^ | Cattle (HF) | 12 | 15 | 17 | 12 | 9.1 | 12 | 6.14.9 | 16 | 6 | **20** | - |
|  | SP-08-NAV-11 | Spain | Navarra^H3^ | Cattle (HF) | 12 | NA | 14 | 11 | 9.1 | 14 | NA | 16 | 6 | **26** | - |
|  | SP-09-MAD-12 | Spain | Madrid^H1^ | Cattle (HF) | 12 | 15 | 17 | NA | 9.1 | 12 | 6.14.9 | 16 | 6 | **20** | - |
|  | SP-10-MAD-13 | Spain | Madrid^H1^ | Cattle (HF) | 13 | 9 | 18 | 12 | 13 | 12 | NA | 16 | 6 | **27** | - |
| 01-F11 | SP-01-GAL-14 | Spain | La Coruña | Cattle (HF) | 13 | 16 | 16 | 12 | 9.1 | 14 | 6.14.9 | 16 | 6 | **28** | [3] |
| 01-F79 | SP-01-GAL-15 | Spain | Lugo | Cattle (HF) | 13 | 15 | NA | NA | 9.1 | 13 | 6.13.10 | 16 | 6 | **29** | - |
| 01-F80 | SP-01-GAL-16 | Spain | Lugo | Cattle (HF) | 12 | 17(I)/18* | 15 | 11 | 9.1 | 14 | 6.13.9 | 16 | 6 | **30** | - |
| 00-F91 | SP-00-GAL-17 | Spain | Lugo | Cattle (HF) | 13 | 17 | 16 | 12 | 9.1 | 15 | 6.14.9 | 16 | 6 | **31** | - |
| F134 | SP-01-GAL-18 | Spain | Lugo | Cattle (HF) | 15 | NA | 14(I)/15* | 12 | 9.1 | NA | 6.14.9 | 16 | 6 | **32** | - |
| F139 | SP-01-GAL-19 | Spain | Lugo | Cattle (HF) | 12 | 16 | 15 | 11 | 9.1 | 15 | 7.13.8 | 15(I)/16* | 6 | **33** | - |
| 02-F152 | SP-02-GAL-20 | Spain | Lugo | Cattle (HF) | 13 | 16 | 15 | 12 | 9.1 | 14 | 6.13.8 | 16 | 6 | **34** | - |
| 01-F186 | SP-01-GAL-21 | Spain | Lugo | Cattle (HF) | 13 | 17 | 16 | 12 | 9.1 | 13 | 6.14.9 | 16 | 6 | **35** | - |
| 01-F187 | SP-01-GAL-22 | Spain | Lugo | Cattle (HF) | 12 | 16 | 15 | 11 | 9.1 | 14 | 6.14.9 | 16 | 6 | **36** | - |
| 01-F189 | SP-01-GAL-23 | Spain | Lugo | Cattle (HF) | 13 | 16 | 14 | 12 | 9.1 | 13 | 6.13.9 | 16 | 6 | **37** | - |
| 02-F200 | SP-02-GAL-24 | Spain | Lugo | Cattle (HF) | 13 | 16 | NA | NA | 9.1 | 13 | 6.14.9 | 16 | 6 | **38** | - |
| 02-F201 | SP-02-GAL-25 | Spain | Lugo | Cattle (HF) | 13 | 16 | NA | NA | 9.1 | 13 | 6.14.9 | 16 | 6 | **38** | - |
| 01-F206 | SP-02-GAL-26 | Spain | Lugo | Cattle (HF) | 13 | 19 | 15 | 12 | 14 | 13 | 6.14.9 | 16 | 6 | **39** | - |
|  | SP-04-GAL-27 | Spain | La Coruña | Cattle (HF) | 13 | 15 | NA | 12 | 9.1 | 12 | 6.13.9 | 17 | 6 | **40** | This study |
|  | SP-04-GAL-28 | Spain | La Coruña^H5^ | Cattle (HF) | 12 | 18 | 17 | 11 | 9.1 | 15 | 6.13.9 | 16 | 6 | **41** | - |
|  | SP-04-GAL-29 | Spain | Pontevedra | Cattle (HF) | 13 | 9 | 13 | 12 | 16 | 11^&^ | NA | 15 | 6 | **42** | - |
|  | SP-04-GAL-30 | Spain | La Coruña^H5^ | Cattle (HF) | 12 | 19 | 17 | 11 | 9.1 | 15 | 6.13.9 | 16 | 6 | **43** | - |
|  | SP-05-GAL-31 | Spain | La Coruña ^H6^ | Cattle (HF) | 11 | 9 | 13 | 11 | 13 | 14 | 6.15.8 | 15 | 6 | **44** | - |
|  | SP-05-GAL-32 | Spain | Lugo^H7^ | Cattle (HF) | NA | 17 | 16 | 13 | 9.1 | 13 | 6.15.9 | 16 | 6 | **45** | - |
|  | SP-05-GAL-33 | Spain | Lugo^H8^ | Cattle (HF) | 14 | 18 | 15 | 13 | 9.1 | 14 | 6.15.9 | 15 | 6 | **46** | - |
|  | SP-05-GAL-34 | Spain | Lugo^H8^ | Cattle (HF) | 13 | 15 | 15 | 11 | 9.1 | 14 | 6.13.9 | 16 | 6 | **47** | - |
|  | SP-05-GAL-35 | Spain | La Coruña^H9^ | Cattle (HF) | 14 | 15 | 16 | 12 | 9.1 | 13 | 7.11.9^&^ | 16 | 6 | **48** | - |
|  | SP-05-GAL-36 | Spain | Lugo^H7^ | Cattle (HF) | 14 | 18 | 16 | 13 | 9.1 | 13 | 6.15.9 | 16 | 6 | **49** | - |
|  | SP-05-GAL-37 | Spain | La Coruña^H9^ | Cattle (HF) | 12 | 11 | NA | NA | 15^&^ | 19 | 6.22.10 | 14^&^ | 6 | **50** | - |
|  | SP-05-GAL-38 | Spain | Lugo | Cattle (HF) | 12 | 16 | 17 | 12 | 9.1 | 13 | 6.15.9 | 16 | 6 | **51** | - |
|  | SP-05-GAL-39 | Spain | Lugo | Cattle (HF) | 14 | 16 | 15 | 12 | 9.1 | 13 | 6.14.10^&^ | 16 | 6 | **52** | - |
|  | SP-05-GAL-40 | Spain | La Coruña | Cattle (HF) | 13 | 15 | 15 | 12 | 9.1 | 13 | 6.14.8 | 16 | 6 | **53** | - |
|  | SP-05-GAL-41 | Spain | La Coruña | Cattle (HF) | 10 | 9 | 13 | 11 | 11 | 12 | 6.20.8^&^ | 14 | 6 | **54** | - |
|  | SP-05-GAL-42 | Spain | Lugo^H7^ | Cattle (HF) | 14 | 18 | 16 | 13 | 9.1 | 13 | 6.15.9 | 16 | 6 | **49** | - |
|  | SP-06-GAL-43 | Spain | La Coruña^H6^ | Cattle (HF) | 11 | 13 | 12 | 11 | 14 | 11 | 6.15.8 | 15 | 6 | **55** | - |
|  | SP-06-GAL-44 | Spain | La Coruña | Cattle (HF) | 12 | 17 | 15 | 12 | 9.1 | 14 | 6.14.9 | 16 | 6 | **56** | - |
|  | SP-06-GAL-45 | Spain | La Coruña^H5^ | Cattle (HF) | 12 | 18 | 17 | 11 | 9.1 | 15 | 6.13.9 | 16 | 6 | **41** | - |
|  | SP-06-GAL-46 | Spain | La Coruña ^H10^ | Cattle (HF) | 12 | 9 | 13 | 11 | 16 | 14 | 6.19.11^&^ | 15 | 6 | **57** | - |
|  | SP-06-GAL-47 | Spain | La Coruña | Cattle (HF) | 13 | 15 | 15 | 12 | 9.1 | 12 | 6.14.9 | 16 | 6 | **58** | - |
|  | SP-06-GAL-48 | Spain | La Coruña | Cattle (HF) | 13 | 14 | 15 | 12 | 9.1 | 13 | 6.14.8 | 16 | 6 | **59** | - |
|  | SP-07-GAL-49 | Spain | La Coruña | Cattle (HF) | 15 | 19 | 15 | 12 | 9.1 | 14 | 6.15.9 | 16 | 6 | **60** | - |
|  | SP-07-GAL-50 | Spain | La Coruña | Cattle (HF) | 13 | 15 | 14 | 12 | 9.1 | 15 | 6.14.9 | 16 | 6 | **61** | - |
|  | SP-07-GAL-51 | Spain | La Coruña | Cattle (HF) | 12 | 12 | 15 | 12 | 9.1 | 14 | 6.14.9 | 16 | 5 | **62** | - |
|  | SP-07-GAL-52 | Spain | La Coruña ^H10^ | Cattle (HF) | 12 | 9 | 13 | 11 | 16 | 11 | 6.19.11 | 16 | 5 | **63** | - |
|  | SP-07-GAL-53 | Spain | La Coruña | Cattle (HF) | 12 | 16 | 15 | 13 | 9.1 | 13 | 6.14.10 | 14 | 6 | **64** | - |
|  | ARG-04-1 | Argentina | Buenos Aires | Cattle | 13 | 17 | 15 | 12 | 9.1 | 13 | 6.15.8 | 16 | 6 | **65** | - |
|  | ARG-04-2 | Argentina | Córdoba | Cattle (HF) | 11 | 10 | 11(I)^&^/10^&,^* | 11 | NA | NA | 6.15.8 | 16 | 5 | **66** | - |
|  | ARG-05-3 | Argentina | Buenos Aires | Cattle | 13 | 10 | NA | 13 | 10 | 14 | 6.12.7^&^ | 16 | 5 | **67** | - |
|  | ARG-05-4 | Argentina | Buenos Aires | Cattle | 13 | 16 | 16 | 12 | 9.1 | 13 | 6.15.9 | 16 | 6 | **68** | - |
|  | ARG-05-5 | Argentina | Buenos Aires | Cattle | 13 | 9 | 13 | 11 | 15 | 16 | 6.17.10^&^ | 16 | 6 | **69** | - |
|  | ARG-05-6 | Argentina | Buenos Aires | Cattle (HF) | 13 | 9 | 13 | 11 | 15 | 16 | 6.17.9^&^ | 16 | 6 | **70** | - |
|  | ARG-05-7 | Argentina | Buenos Aires | Cattle | 13 | 9 | 13 | 11 | 14 | 13 | 6.18.10 | 16 | 6 | **71** | - |
|  | ARG-05-8 | Argentina | Buenos Aires | Cattle | 13 | NA | 16 | 12 | 9.1 | 13 | 5.15.9 | 16 | 6 | **72** | - |
|  | ARG-07-9 | Argentina | Santa Fe | Cattle | 13 | 9 | 14 | 11 | 14 | 16 | 6.17.11^&^ | 16 | 6 | **73** | - |
|  | ARG-07-10 | Argentina | Buenos Aires | Cattle | 11 | 14 | 15 | 12 | 9.1 | 12 | 6.15.9 | 16 | 6 | **74** | - |
|  | ARG-07-11 | Argentina | Buenos Aires | Cattle | 13 | 11 | NA | NA | NA | 16 | 6.16.9 | 16 | 6 | **75** | - |
|  | ARG-07-12 | Argentina | Buenos Aires | Cattle | 13 | 9 | 13 | 11 | 16 | 16 | 6.18.10 | 16 | 6 | **76** | - |
|  | ARG-07-13 | Argentina | Buenos Aires | Cattle | 11 | NA | 14 | 12 | NA | 12 | 6.15.9 | 16 | NA | **77** | - |
|  | ARG-08-14 | Argentina | Buenos Aires | Cattle (HF) | 13 | 9 | 16 | 12 | 16 | 13 | 6.15.9 | 16 | 6 | **78** | - |
|  | ARG-08-15 | Argentina | Buenos Aires | Cattle | 12 | 15 | 14 | 12 | 9.1 | 12 | 6.15.9 | 16 | 6 | **79** | - |
|  | ARG-08-16 | Argentina | Buenos Aires | Cattle | 13 | 14 | NA | 12 | NA | 12 | 6.15.9 | 16 | 6 | **80** | - |
|  | SCOT-07-1 | Scotland | Galloway |  | 10 | NA | 15 | 12 | 12 | 12 | 6.25.9 | 16 | 6 | **81** | - |
|  | SCOT-08-2 | Scotland | Galloway |  | 16^&^ | 9 | 23^&^ | 12 | 13 | NA | 6.25.10^&^ | 16 | 6 | **82** | - |
|  | SCOT-08-3 | Scotland | Galloway |  | 13 | 17 | 15 | 12 | 10.1^&^ | 12 | 6.12.8^&^ | 16 | 6 | **83** | - |
|  | SCOT-08-4 | Scotland | Galloway |  | 11 | 10 | 13 | 11 | 9.1 | 17 | 6.16.8 | 17 | 6 | **84** | - |
|  | SCOT-08-5 | Scotland | Galloway |  | 11 | 10 | 13 | 11 | 9.1 | 16 | 6.15.8 | 17 | 6 | **85** | - |
|  | SCOT-08-6 | Scotland | Galloway |  | 13 | 9 | 13 | 11 | 15 | 11 | 6.20.10 | 15 | 6 | **86** | - |
|  | SCOT-08-7 | Scotland | Galloway |  | 11 | NA | 13(I)/12* | 11 | 9.1 | 17 | NA | 17 | 6 | **84** | - |
|  | SCOT-08-8 | Scotland | Galloway |  | 11 | 10 | NA | NA | 9.1 | 18^&^ | 6.17.8 | 17 | NA | **87** | - |
|  | SCOT-08-9 | Scotland | Galloway |  | 11 | 9 | 13 | 11 | 9.1 | 17 | 6.15.8 | 16 | 6 | **88** | - |
|  | GER-00-1 | Germany | Lower Saxony | Cattle (HF) | 15 | 13 | 13 | 12 | 19^&^ | 16 | 6.22.10 | 15 | 6 | **89** | - |
|  | GER-08-2 | Germany | Northrhine-Westphalia | Cattle (HF) | 13 | 13 | 15 | 12 | 9.1 | 16 | 6.14.8 | 16 | 6 | **90** | - |
|  | GER-08-3 | Germany | Schleswig-Holstein | Cattle (HF) | 13 | 17 | 15 | 12 | 11 | NA | 6.13.9 | 16 | 6 | **91** | - |
|  | GER-08-4 | Germany | Rhineland-Palatinate | Cattle (HF) | 11 | 11 | 13 | 11 | 9.1 | 16 | 6.15.8 | 17 | 6 | **92** | - |
|  | GER-09-5 | Germany | Brandenburg | Cattle (HF) | 15 | 15 | 13 | 12 | 18 | 13 | 6.19.10 | 15 | 6 | **93** | - |
|  | GER-05-6 | Germany | Bavaria | Cattle (HF) | 11 | 9 | 13 | 11 | 13 | 14 | 6.15.8 | 15 | 6 | **44** | - |
|  | GER-05-7 | Germany | Bavaria | Cattle (HF) | 12 | 9 | 13 | 13 | 11 | NA | 7.17.9 | 17 | 6 | **94** | - |
|  | GER-09-8 | Germany | Lower Saxony | Cattle (HF) | 15 | 12 | 15 | 12 | 18 | 16 | 6.14.8 | 14 | 6 | **95** | - |
|  | GER-09-9 | Germany | Hesse | Cattle (HF) | 14 | NA | NA | 13 | 12 | 20^&^ | 5.22.9^&^ | 15 | 6 | **96** | - |

^a^ Sample identification in previous studies [1-4].

^b^ Sample identification in this study: SP (Spain), ARG (Argentina), SCOT (Scotland), GER (Germany) - year of sample collection- MAD (Madrid), GAL (Galicia), NAV (Navarra), ZAR (Zaragoza), LEON (León), VAS (País Vasco).

^c^ Allele polymorphism of MS markers are expressed as the number of repeats (Table 1).

^d^ Note -: Identical to the previous reference (located above).

^e^ MLG: Multilocus microsatellite genotype (number).

NA: not amplified, not assignated.

^Hnº^: Hnº superscript denotes identical herd origin for samples.

^#^ HF denotes Holstein-Friesian breed.

* Two alleles were observed for these MS markers. Only, the predominant allele (I) was included in further analysis.

^&^ Newly described MS alleles in this study.

^§^ NC-1 samples from different research laboratories were genotyped; NC-1 DNA sample extracted in SALUVET Group of University Complutense of Madrid (Spain) from the NC-1 isolate that was kindly provided by Dr. Boothroyd (Department of Microbiology and Immunology, Stanford University School of Medicine, USA), NC-1 DNA extracted in Centro de Investigaciones Agrarias of Mabegondo (Spain) from the NC-1 isolate that was also kindly provided by Dr. Boothroyd, the NC-1 DNA extracted in Friedrich-Loeffler-Institut of Wusterhausen (Germany), the NC-1 DNA sample extracted in Consejo Nacional de Investigaciones Científicas y Técnicas (CONICET) of Balcarce (Argentina), the NC-1 DNA kindly provided by Dr. Venturini (Inmunoparasitology and Parasitology Laboratory, National University of La Plata, Argentina), the NC-1 DNA kindly provided by Dr. Barragan (Center for Infectious Medicine, Karolinska Institutet, Sweden) and the NC-1 DNA kindly provided by Dr. Hemphill (Institute of Parasitology, University of Berne, Switzerland). Multilocus microsatellite genotype of the NC-1 DNA sample extracted in Balcarce (Argentina) showed MS6B allele with one repeat unit less (16).

Microsatellites labelled in grey indicate seven-loci microsatellite genotypes (MLGs) involved in data analyses.

**References**

1. Regidor-Cerrillo J, Pedraza-Diaz S, Gomez-Bautista M, Ortega-Mora LM (2006) Multilocus microsatellite analysis reveals extensive genetic diversity in *Neospora caninum*. J Parasitol 92: 517-524.

2. Regidor-Cerrillo J, Gomez-Bautista M, Pereira-Bueno J, Aduriz G, Navarro-Lozano V, et al. (2008) Isolation and genetic characterization of *Neospora caninum* from asymptomatic calves in Spain. Parasitology 135: 1651-1659.

3. Pedraza-Diaz S, Marugan-Hernandez V, Collantes-Fernandez E, Regidor-Cerrillo J, Rojo-Montejo S, et al. (2009) Microsatellite markers for the molecular characterization of *Neospora caninum*: Application to clinical samples. Vet Parasitol 166: 38-46.

4. Moreno B, Collantes-Fernandez E, Villa A, Navarro A, Regidor-Cerrillo J, et al. (2012) Occurrence of *Neospora caninum* and *Toxoplasma* *gondii* infections in ovine and caprine abortions. Vet Parasitol 187: 312-318.
