# Supplementary material for: Genetic Diversity and Geographic Population Structure of Bovine Neospora caninum Determined by Microsatellite Genotyping Analysis
Source: PLoS One. 2013 Aug 6;8(8):e72678. doi: 10.1371/journal.pone.0072678 (PMC3735528; doi:10.1371/journal.pone.0072678)
Supplement: Table S2 — (DOCX) [file pone.0072678.s004.docx]

**Supplementary Table 2:** Chromosomal location and summary of the alleles for each microsatellite marker.

| **Marker** | **Microsatellite sequence** | **Repeated motif** | **Length of repeat^a^** | **Allele number^b^** | **Length of amplicon (bp)^c^** | **Chromosomal location^d^** |
| --- | --- | --- | --- | --- | --- | --- |
| **MS4** | GC*-(AT)_n_-ACATTT-(AT)_2_*-AC | (AT)_n_ | 15 | 10 | 294 | **NCLIV_chrIX (5.49Mb)** |
|  |  |  | 16 | 11 | 296 | (1988484-1988528) |
|  |  |  | 17 | 12 | 298 |  |
|  |  |  | 18 | 13 | 300 |  |
|  |  |  | 19 | 14 | 302 |  |
|  |  |  | 20 | 15 | 304 |  |
|  |  |  | 21 | 16 | 306 |  |
| **MS5** | CG*-(TA)_n_-TG-TA*-GG | (TA)_n_ | 9 | 7^#^ | 300 | **NCLIV_chrIX (5.49Mb)** |
|  |  |  | 11 | 9 | 304 | (779604-779642) |
|  |  |  | 12 | 10 | 306 |  |
|  |  |  | 13 | 11 | 308 |  |
|  |  |  | 14 | 12 | 310 |  |
|  |  |  | 15 | 13 | 312 |  |
|  |  |  | 16 | 14 | 314 |  |
|  |  |  | 17 | 15 | 316 |  |
|  |  |  | 18 | 16 | 318 |  |
|  |  |  | 19 | 17 | 320 |  |
|  |  |  | 20 | 18 | 322 |  |
|  |  |  | 21 | 19 | 324 |  |
| **MS6A** | GC*-(TA)_n_-*AC | (TA)_n_ | 10 | 10^#^ | 294 | **NCLIV_chrX (6.99Mb)** |
|  |  |  | 11 | 11 | 296 | (5563546-5563513) |
|  |  |  | 12 | 12 | 298 |  |
|  |  |  | 13 | 13 | 300 |  |
|  |  |  | 14 | 14 | 302 |  |
|  |  |  | 15 | 15 | 304 |  |
|  |  |  | 16 | 16 | 306 |  |
|  |  |  | 17 | 17 | 308 |  |
|  |  |  | 18 | 18 | 310 |  |
|  |  |  | 19 | 19 | 312 |  |
|  |  |  | 23 | 23 | 320 |  |
| **MS6B** | CC*-(AT)_n_-*GT | (AT)_n_ | 11 | 11 | 287 | **NCLIV_chrX (6.99Mb)** |
|  |  |  | 12 | 12 | 289 | (5563338-5563311) |
|  |  |  | 13 | 13 | 291 |  |
|  |  |  | 14 | 14 | 293 |  |
|  |  |  | 17 | 17 | 299 |  |
| **MS7** | *AT*-TA-(TA)_n_-*GG | (TA)_n_*^SNP^ | 10* | 9.1 | 279 | [**NCLIV_chrVIIa**](http://toxodb.org/toxo/showRecord.do?name=SequenceRecordClasses.SequenceRecordClass&project_id=ToxoDB&primary_key=NCLIV_chrVIIa) (**3.95Mb)** |
|  |  |  | 11* | 10.1 | 281 | (2535410-2535443) |
|  | AT-*AA-(TA)_n_*-GG | (TA)_n_ | 11 | 10 | 281 |  |
|  |  |  | 12 | 11 | 283 |  |
|  |  |  | 13 | 12 | 285 |  |
|  |  |  | 14 | 13 | 287 |  |
|  |  |  | 15 | 14 | 289 |  |
|  |  |  | 16 | 15 | 291 |  |
|  |  |  | 17 | 16 | 293 |  |
|  |  |  | 19 | 18 | 297 |  |
|  |  |  | 20 | 19 | 299 |  |
| **MS8** | TGAC*-(AT)_n_-*GG | (AT)_n_ | 11 | 11 | 285 | **NCLIV_chrII (2.17Mb)** |
|  |  |  | 12 | 12 | 287 | (138005-137965) |
|  |  |  | 13 | 13 | 289 |  |
|  |  |  | 14 | 14 | 291 |  |
|  |  |  | 15 | 15 | 293 |  |
|  |  |  | 16 | 16 | 295 |  |
|  |  |  | 17 | 17 | 297 |  |
|  |  |  | 18 | 18 | 299 |  |
|  |  |  | 19 | 19 | 301 |  |
|  |  |  | 20 | 20 | 303 |  |

**Supplementary Table 2** (Continued).

| **MS10** | AGT*-(ACT)_x_-(AGA)_y_-(TGA)_z_-*CAA | (ACT)_x_-(AGA)_y_-(TGA)_z_ | 5/14/9 | 5.14.9 | 298 | **NCLIV_chrVIII (6.72Mb)** |
| --- | --- | --- | --- | --- | --- | --- |
|  |  |  | 5/15/9 | 5.15.9 | 301 | (5227071-5227205) |
|  |  |  | 5/22/9 | 5.22.9 | 322 |  |
|  |  |  | 6/12/7 | 6.12.7 | 289 |  |
|  |  |  | 6/12/8 | 6.12.8 | 292 |  |
|  |  |  | 6/13/8 | 6.13.8 | 295 |  |
|  |  |  | 6/13/9 | 6.13.9 | 298 |  |
|  |  |  | 6/13/10 | 6.13.10 | 301 |  |
|  |  |  | 6/14/8 | 6.14.8 | 298 |  |
|  |  |  | 6/14/9 | 6.14.9 | 301 |  |
|  |  |  | 6/14/10 | 6.14.10 | 304 |  |
|  |  |  | 6/15/8 | 6.15.8 | 301 |  |
|  |  |  | 6/15/9 | 6.15.9 | 304 |  |
|  |  |  | 6/16/8 | 6.16.8 | 304 |  |
|  |  |  | 6/16/9 | 6.16.9 | 307 |  |
|  |  |  | 6/17/8 | 6.17.8 | 307 |  |
|  |  |  | 6/17/9 | 6.17.9 | 310 |  |
|  |  |  | 6/17/10 | 6.17.10 | 313 |  |
|  |  |  | 6/17/11 | 6.17.11 | 316 |  |
|  |  |  | 6/18/10 | 6.18.10 | 316 |  |
|  |  |  | 6/19/10 | 6.19.10 | 319 |  |
|  |  |  | 6/19/11 | 6.19.11 | 321 |  |
|  |  |  | 6/20/8 | 6.20.8 | 316 |  |
|  |  |  | 6/20/10 | 6.20.10 | 322 |  |
|  |  |  | 6/21/10 | 6.21.10 | 325 |  |
|  |  |  | 6/22/9 | 6.22.9 | 325 |  |
|  |  |  | 6/22/10 | 6.22.10 | 328 |  |
|  |  |  | 6/23/10 | 6.23.10 | 331 |  |
|  |  |  | 6/24/8 | 6.24.8 | 328 |  |
|  |  |  | 6/25/9 | 6.25.9 | 334 |  |
|  |  |  | 6/25/10 | 6.25.10 | 337 |  |
|  |  |  | 6/26/10 | 6.26.10 | 340 |  |
|  |  |  | 7/11/9 | 7.11.9 | 295 |  |
|  |  |  | 7/12/9 | 7.12.9 | 298 |  |
|  |  |  | 7/13/8 | 7.13.8 | 298 |  |
|  |  |  | 7/17/9 | 7.17.9 | 313 |  |
|  |  |  | 8/23/8 | 8.23.8 | 331 |  |
| **MS12** | GC*-(GT)_n_-*GC | (GT)_n_ | 14 | 14 | 304 | [**NCLIV_chrXII**](http://toxodb.org/toxo/showRecord.do?name=SequenceRecordClasses.SequenceRecordClass&project_id=ToxoDB&primary_key=NCLIV_chrXII) (**6.47Mb)** |
|  |  |  | 15 | 15 | 306 | (3561796-3561761) |
|  |  |  | 16 | 16 | 308 |  |
|  |  |  | 17 | 17 | 310 |  |
| **MS21** | TG*-(TACA)_3_-TACC-(TACA)_n_-*TT | (TACA)_n_ | 9 | 5 | 299 | [**NCLIV_chrVIIa**](http://toxodb.org/toxo/showRecord.do?name=SequenceRecordClasses.SequenceRecordClass&project_id=ToxoDB&primary_key=NCLIV_chrVIIa) **(3.95Mb)** |
|  |  |  | 10 | 6 | 303 | (1931759-1931803) |

^a^ Length of repeat established in [1]. Repeated motif sequences are indicated by italics in the MS sequence.

^b^ Allele number as the number of repeats assigned in this study.

^c^ Length of amplicon according to the MS sequence [1].

^d^ Size of chromosome and position of the MS sequence determined for the Nc-Liv isolate in the ToxoDB database (ToxoDB database; http://toxodb.org/toxo/).

* Alleles with a single nucleotide polymorphism.

^#^ Alleles identified as secondary in the electropherogram based on fragment size analysis.

**Reference**

1. Regidor-Cerrillo J, Pedraza-Diaz S, Gomez-Bautista M, Ortega-Mora LM (2006) Multilocus microsatellite analysis reveals extensive genetic diversity in *Neospora caninum*. J Parasitol 92: 517-524.
