# Supplementary material for: Genetic Diversity and Geographic Population Structure of Bovine Neospora caninum Determined by Microsatellite Genotyping Analysis
Source: PLoS One. 2013 Aug 6;8(8):e72678. doi: 10.1371/journal.pone.0072678 (PMC3735528; doi:10.1371/journal.pone.0072678)
Supplement: Table S3 — (DOCX) [file pone.0072678.s005.docx]

**Supplementary Table 3:** Genetic diversity in *N. caninum* by locus and geographic population (country).

|  | ***POPULATION***  **(Country)** | ***LOCI*** | | | | | | | **Mean ± SD** |
| --- | --- | --- | --- | --- | --- | --- | --- | --- | --- |
|  |  | **MS4** | **MS5** | **MS6A** | **MS7** | **MS8** | **MS10** | **MS12** |  |
| **Number alleles per locus (A)** | Worldwide | 6 | 8 | 5 | 5 | 7 | 11 | 3 | **6.42 ± 2.57** |
|  | Spain | 6 | 11 | 7 | 8 | 7 | 17 | 4 | **8.57 ± 4.28** |
|  | Argentina | 3 | 7 | 5 | 5 | 4 | 9 | 1 | **4.86± 2.61** |
|  | Scotland | 4 | 3 | 3 | 5 | 5 | 7 | 3 | **4.29 ± 1.50** |
|  | Germany | 5 | 6 | 2 | 6 | 4 | 7 | 4 | **4.86± 1.68** |
| **Allele richness per locus (Ar)** | Worldwide | 5.63 | 7.37 | 4.75 | 4.86 | 6.50 | 9.67 | 2.87 | **5.95 ± 2.17** |
|  | Spain | 4.14 | 6.69 | 5.52 | 3.56 | 5.23 | 7.14 | 2.79 | **5.01 ± 1.60** |
|  | Argentina | 2.67 | 5.94 | 4.76 | 4.78 | 3.72 | 6.98 | 1.00 | **4.26 ± 2.01** |
|  | Scotland | 3.92 | 3.00 | 2.99 | 4.84 | 4.98 | 6.95 | 2.96 | **4.23 ± 1.48** |
|  | Germany | 4.92 | 5.97 | 2.00 | 5.88 | 4.00 | 6.80 | 3.96 | **4.79 ± 2.05** |
| **Genetic diversity (*He*)** | Worldwide | 0.83 | 0.90 | 0.78 | 0.81 | 0.87 | 0.95 | 0.45 | **0.80 ± 0.16** |
|  | Spain | 0.68 | 0.84 | 0.80 | 0.35 | 0.80 | 0.83 | 0.37 | **0.67 ± 0.21** |
|  | Argentina | 0.41 | 0.78 | 0.80 | 0.77 | 0.73 | 0.83 | 0 | **0.62 ± 0.31** |
|  | Scotland | 0.66 | 0.66 | 0.57 | 0.68 | 0.80 | 0.90 | 0.63 | **0.70 ± 0.11** |
|  | Germany | 0.81 | 0.87 | 0.50 | 0.86 | 0.66 | 0.89 | 0.73 | **0.76 ± 0.14** |
